# Role of METTL6 in gastric cancer: diagnostic, prognostic, and functional implications

**DOI:** 10.7717/peerj.21763

**Published:** 2026-11-02

**Authors:** Dongdong Lu, Jie Lou, Leijie Huang, Chunyan Weng

**Affiliations:** 1 Department of Gastroenterology, Ningbo No.2 Hospital, Ningbo, China; 2 Department of Gastroenterology, The First Affiliated Hospital of Zhejiang Chinese Medical University (Zhejiang Provincial Hospital of Chinese Medicine), Hangzhou, China

**Keywords:** Gastric cancer, METTL6, Prognosis, Survival analysis

## Abstract

**Background:**

Gastric cancer (GC) remains among the most common and lethal malignancies worldwide. The identification of novel biomarkers is critical for improving prognostic evaluation and informing therapeutic decision-making. Methyltransferase-like 6 (METTL6), a transfer RNA methyltransferase implicated in cell division, has a poorly defined biological role and mechanism in GC.

**Methods:**

METTL6 expression was systematically investigated using experimental techniques and bioinformatic analyses. Comprehensive clinical and pathological data were collected to stratify patients with GC for comparative evaluation. Kaplan–Meier survival analysis and Cox proportional hazards regression models were applied to determine the prognostic significance of METTL6 expression. In parallel, immune cell infiltration associated with METTL6 expression was analyzed using data from The Cancer Genome Atlas, and the prognostic impact of METTL6 was further assessed within immune infiltration–defined subgroups. METTL6 knockdown was achieved through lentiviral transduction, and its biological effects were examined both * in vitro* and * in vivo* using colony formation, wound healing, Transwell migration and invasion assays, and xenograft mouse models.

**Results:**

METTL6 was markedly overexpressed in GC cell lines. Elevated METTL6 expression was significantly associated with sex, clinical stage, histological type, histological grade, and poorer overall survival. In addition, METTL6 expression demonstrated a strong correlation with immune cell infiltration. Functional experiments showed that METTL6 knockdown significantly inhibited colony formation, wound closure, and tumor cell migration and invasion *in vitro*. Consistently, METTL6 silencing substantially reduced tumor volume and tumor weight in xenograft-bearing mice.

**Conclusion:**

METTL6 overexpression is closely associated with an unfavorable prognosis in GC and may represent a valuable prognostic biomarker. Furthermore, METTL6 warrants further investigation as a potential therapeutic target.

## Introduction

Gastric cancer (GC) represents a major global health burden, ranking as the fifth most prevalent malignancy and a leading cause of cancer-related mortality (Bray et al., 2024). GC is frequently diagnosed at an advanced stage because of its insidious onset and nonspecific clinical manifestations, often resulting in local or distant metastasis (Muro et al., 2019). Although multiple therapeutic strategies for GC are currently available, their effectiveness is frequently limited by late-stage diagnosis and the pronounced heterogeneity of the tumor, which contributes to variable treatment responses. Therefore, further investigation into reliable diagnostic and prognostic biomarkers for GC development and progression is urgently needed.

Methyltransferase-like proteins (METTLs) constitute a large protein family characterized by S-adenosylmethionine–binding domains (Ignatova et al., 2019). Members of the METTL family participate in diverse cellular processes and play important roles in tumor progression (Chen et al., 2022; Davydova et al., 2021; Jiang et al., 2021; Peng et al., 2022; Rong et al., 2020; Wang et al., 2020; Zeng et al., 2020). As a member of the METTL family, the biological function of methyltransferase-like 6 (METTL6) has recently begun to be elucidated. For instance, METTL6 is overexpressed in tumor tissues, such as breast cancer tissues (Gatza et al., 2014), and its knockout enhances cisplatin sensitivity in lung cancer cells (Tan et al., 2011). Moreover, METTL6 loss impairs mouse stem cell pluripotency, suggesting that METTL6 is a critical regulator of hepatocellular carcinoma cell proliferation (Ignatova et al., 2020). Mechanistically, METTL6 catalyzes the methylation of cytidine at position C32 in specific serine transfer RNA isoacceptors, thus regulating tumor proliferation, stem cell self-renewal, and metabolic activity, highlighting its potential as a therapeutic target in cancer (Ignatova et al., 2020). This modification further influences the translation of genes involved in cell cycle control and DNA repair, particularly those enriched in serine AGU codons, thereby affecting tumor growth, cell cycle progression, and cellular responses to DNA damage (Cui et al., 2024). Notably, a recent study established a prognostic scoring system based on five genes, including METTL6, demonstrating that high expression of these genes predicted poor outcomes in hepatocellular carcinoma (Dai et al., 2021). However, despite growing evidence implicating METTL6 in multiple malignancies, its expression profile, clinical relevance, and functional role in GC have not been systematically investigated. In particular, the prognostic value of METTL6 in GC and its relationship with the tumor immune microenvironment remain largely unknown.

In the present study, METTL6 expression was examined using a combination of experimental assays and computational analyses. Moreover, the associations among METTL6 overexpression, clinicopathological features, and clinical outcomes in patients with GC were systematically evaluated. We also identified a significant correlation between METTL6 expression and immune cell infiltration.

## Methods

### XIANTAO platform

Data on METTL6 expression and corresponding prognostic outcomes were obtained from the XIANTAO platform (Chen et al., 2023a; Gao et al., 2022), which is based on R and the ggplot2 R package. A nomogram was constructed based on METTL6 expression levels and clinicopathological variables derived from the XIANTAO platform. In addition, the “Immune Infiltration” module was used to evaluate the associations between immune cell populations and METTL6 expression.

### Genetic alteration analysis

The cBioPortal web platform (https://www.cbioportal.org/results/cancerTypesSummary?case_set_id=all&gene_list=METTL6&cancer_study_list=5c8a7d55e4b046111fee2296) (Cerami et al., 2012) was used to examine the frequency, copy number alterations, and mutation profiles of METTL6 across The Cancer Genome Atlas (TCGA) Pan-Cancer Atlas studies.

### Analysis of databases using the Kaplan–Meier plotter

The prognostic significance of METTL6 in GC was evaluated using the Kaplan–Meier Plotter (https://kmplot.com/analysis/index.php?p=service&cancer=gastric) (Győrffy, 2021). The median METTL6 expression value was used as the cutoff to stratify patients into high- and low-expression groups, followed by comparative analyses of first progression (FP), overall survival (OS), and post-progression survival (PPS).

### Interacting and correlated genes of METTL6

The STRING database (https://string-db.org/network/9606.ENSP00000407613) (Szklarczyk et al., 2021) was used to construct the METTL6 protein–protein interaction network. Specifically, the minimum required interaction score was set to medium confidence (0.4), and the maximum number of interactors displayed in the first shell was limited to 50, while all other parameters were maintained at default settings. The top 100 genes most strongly associated with METTL6 were identified. The expression relationships between METTL6 and the three most highly correlated genes were further analyzed using GEPIA2 (http://gepia.cancer-pku.cn/detail.php?gene=METTL6) (Tang et al., 2019).

### Tumor immune estimation resource

The TIMER web server (Li et al., 2016; Li et al., 2017; Li et al., 2020) was used to analyze differences in immune cell infiltration between tumor tissues with high and low METTL6 expression across 32 different cancer types. The “TIMER-Gene” and “Immune-Gene” modules were applied to examine the relationships between METTL6 levels and immune cell infiltration in cancers. All online platforms (XIANTAO, TIMER2.0, Kaplan–Meier Plotter, GEPIA2, STRING, and cBioPortal) were accessed in January 2025.

### Cell culture and transfection

Human GC cell lines MKN-74 and MKN-1 and the human normal gastric epithelial cell line GES-1 were obtained from the American Type Culture Collection. All cells were cultured in RPMI-1640 medium supplemented with 10% fetal bovine serum and maintained at 37 °C in a humidified atmosphere containing 5% CO2. Cell line identity and quality control information (authentication and mycoplasma status) were obtained from the vendor at purchase. All experiments were conducted using cells within a limited number of passages after thawing. For gene silencing, METTL6 knockdown and negative control lentiviral particles were generated by Genechem (Shanghai, China). Cell infection was performed as previously described (Hu et al., 2025). The sequences of the short hairpin RNA (shRNA) targeting METTL6 were as follows: shMETTL6-1#, 5′-TTAAGTATAAAAAATATCTTCC-3′; shMETTL6-2#, 5′-TTAAATTCCTACATGATATTTA-3′; and negative control shRNA, 5′-GCGCGATA- GCGCTAATAATTT-3′.

### Western blot

The cells were lysed to extract total protein according to standard protocols. Protein samples were loaded onto gels and transferred onto membranes, which were subsequently blocked with serum-free block solution for 15 min. The membranes were incubated overnight at 4 °C with anti-METTL6 antibody (15120-1-AP; Proteintech) and anti-vinculin antibody (ET1705-94; HUABIO). After washing, the membranes were incubated with HRP-linked rabbit or mouse antibody for 1.5 h at 25 °C. Protein signals were detected using an enhanced chemiluminescence (ECL) detection system. METTL6 antibody specificity was confirmed by the marked reduction of METTL6 signal following shRNA-mediated knockdown.

### Colony formation

A total of 1 × 10^3^ cells were seeded into 35 mm culture dishes. After 2 weeks of incubation, colonies were fixed with methanol and stained with crystal violet. Colony numbers were quantified using ImageJ software (USA).

### Wound healing assay

A total of 8 × 10^4^ MKN-74 and MKN-1 cells were seeded into the inserts of wound healing assay chambers (ibidi, Germany). After 24 h, the inserts were removed, and the cells were cultured in serum-free medium. Wound closure was evaluated by measuring scratch widths at 0 h and 36 h using light microscopy.

### Transwell assays

Following the manufacturer’s instructions, cell migration and invasion assays were performed using 8-µm pore size Transwell chambers (Costar), and Matrigel-coated chambers (Kennebunk, ME, USA) were used for invasion assays. A total of 3 × 10^4^ and 5 × 10^4^ MKN-74 cells were seeded into the upper chambers and incubated for 48 h. Cells on the lower surface were then fixed, stained, and counted.

### Vertebrate animal study methods

Male BALB/c nude mice aged 4–6 weeks were obtained from Hangzhou Qizhen Animal Company and maintained under specific pathogen-free conditions. Animals were housed under standard environmental conditions with controlled temperature and humidity, provided ad libitum access to food and water, and supplied with environmental enrichment to promote animal welfare. All procedures were conducted in accordance with approved experimental protocols. Analgesia was administered for invasive procedures but was withheld for minor interventions when necessary to avoid potential confounding effects. Behavioral, physiological, and histological outcomes were systematically evaluated. Sampling was minimally invasive; anesthesia was administered when required, and its omission for noninvasive techniques was scientifically justified. Mice were monitored every day for signs of pain, distress, or abnormal behavior. Humane endpoints were predefined as a body weight loss exceeding 20%, severe lethargy, inability to obtain food or water, or ulcerated tumors more than 1.5 cm in diameter. Any animal reaching these criteria was immediately euthanized using the method described below. During the course of the study, no mice met the humane endpoint criteria before the scheduled termination.

At the conclusion of the experiment, all surviving animals were humanely euthanized by CO_2_ inhalation for tissue collection and subsequent analyses. CO_2_ was introduced into the chamber at a controlled rate of 30–40% of the chamber volume per minute to confirm a gradual increase in concentration. After loss of consciousness, the flow rate was increased to achieve 100% CO_2_ concentration to ensure death. Cessation of respiration and heartbeat was established before removal from the chamber. All tumor diameters remained below two cm throughout the study.

Experimental units were randomly assigned to control and treatment groups using a random number generator. Potential confounders, including treatment order, measurement sequence, and cage location, were minimized by balanced allocation and random rotation across groups. Group allocation was known only to the investigator responsible for assignment. Personnel conducting the experiments, assessing outcomes, and analyzing the data were blinded to group identity. No *a priori* inclusion or exclusion criteria were applied, and all animals, experimental units, and data points were included in the final analysis. The final sample size was *n* = 6 per group.

A total of 18 mice were used in this study, with six animals allocated to each group. Sample size determination was based on pilot data. When mice reached 6 weeks of age, they were subcutaneously injected in the axillary region with either shMETTL6- or shCtrl-transduced MKN-74 cells. Tumor length and width were measured weekly, and tumor volume was calculated as follows: volume = length × width^2^/2. All tumor diameters and volumes are reported in Table S1. At the end of the experiment, tumors were excised, photographed, and weighed. All animal experiments were conducted in accordance with institutional and national guidelines and were approved by the Ethics Committee of Zhejiang Chinese Medical University (IACUC-20230424-04).

### Statistical analysis

Statistical analyses for the *in vitro* and *in vivo* experimental validation were performed using SPSS version 25.0 (IBM Corp., Armonk, NY, USA). Each *in vitro* experiment was independently repeated at least three times, and data are presented as the mean ± standard deviation (SD). Comparisons between two groups were performed using Student’s *t*-test, whereas comparisons among multiple groups were conducted using one-way analysis of variance (ANOVA) followed by Tukey’s *post-hoc* test, as appropriate. GraphPad Prism version 9.00 (GraphPad Software Inc., San Diego, CA, USA) was used exclusively for graphical presentation of the experimental data.

For analyses based on publicly available transcriptomic and clinical datasets, computational analyses were performed using R (version 4.2.1; R Core Team, 2022) and the corresponding online bioinformatics platforms. Clinicopathological comparisons were performed using the Wilcoxon rank-sum test, one-way ANOVA, or the Kruskal–Wallis test, as appropriate. Survival differences were evaluated using the log-rank (Mantel–Cox) test, and hazard ratios (HRs) with 95% confidence intervals (CIs) were estimated using univariate Cox proportional hazards regression models implemented through the XIANTAO survival module. Detailed statistical tests and software used for individual figure panels are provided in Table S3. A two-sided *p* value < 0.05 was considered statistically significant.

## Results

### Gene expression data analysis in GC

METTL6 mRNA expression was evaluated across 33 cancer types using pan-cancer datasets from TCGA and Genotype-Tissue Expression projects *via* the XIANTAO online platform. Elevated METTL6 expression was observed in 15 cancer types (Fig. 1A). Notably, METTL6 levels were significantly higher in stomach adenocarcinoma (STAD) tissues than in normal gastric tissues (Fig. 1B). Consistently, analysis of 27 matched tumor and adjacent normal tissue pairs confirmed significantly increased METTL6 expression in tumor specimens (Fig. 1C). Receiver operating characteristic curve analysis yielded a 1-year area under the curve of 0.822, indicating strong diagnostic potential (Fig. 1D). These results suggest that METTL6 serves as a promising biomarker in GC. To further validate the role of METTL6 in GC, an additional seven paired tumor and adjacent normal tissue samples were examined. Western blot analysis demonstrated increased METTL6 protein levels in tumor tissues (Fig. 1E). Furthermore, cBioPortal OncoPrint analysis was used to investigate genomic alterations of METTL6 in STAD cases from the TCGA dataset. The results showed that METTL6 exhibited a low mutation frequency, with gene amplification, deep deletions, truncating mutations, and missense mutations collectively occurring in fewer than 2% of cases (Fig. 1F).

**Figure 1:**
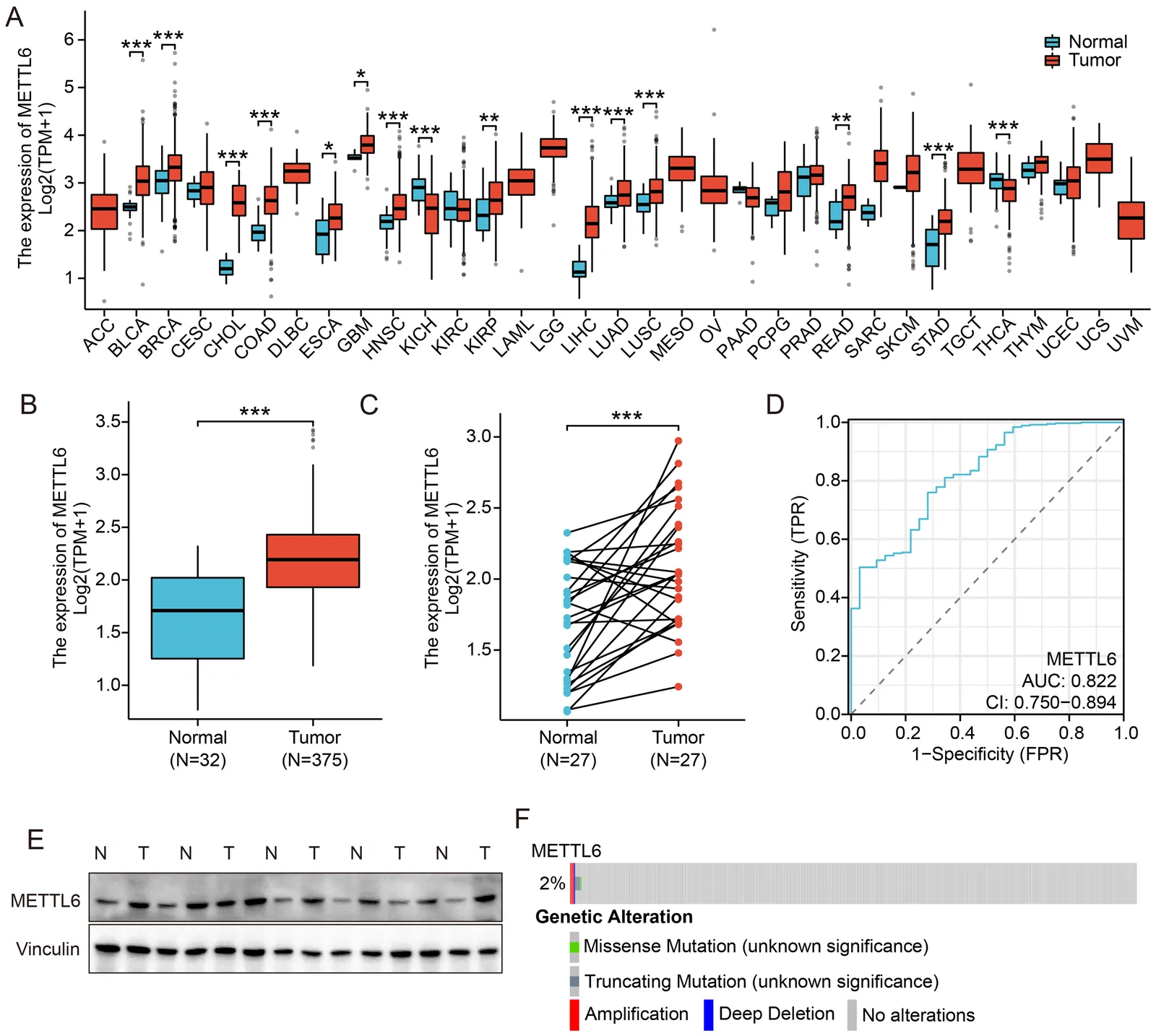
Methyltransferase-like 6 (METTL6) is overexpressed in gastric cancer (GC). (A) METTL6 expression across multiple cancer types based on The Cancer Genome Atlas (TCGA) and Genotype-Tissue Expression data. (B and C) METTL6 expression in tumor tissues and paired adjacent normal tissues from patients with GC in the TCGA dataset. (D) Diagnostic performance of METTL6 in GC. (E) METTL6 protein expression in cancerous and adjacent non-cancerous tissues. (F) cBioPortal OncoPrint showing METTL6 genomic alterations in patients with GC. Abbreviation: Adrenocortical carcinoma (ACC), Bladder urothelial carcinoma (BLCA), Breast invasive carcinoma (BRCA), Cervical squamous cell carcinoma and endocervical adenocarcinoma (CESC), Cholangiocarcinoma (CHOL), Colon adenocarcinoma (COAD), Lymphoid neoplasm diffuse large B-cell lymphoma (DLBC), Esophageal carcinoma (ESCA), Glioblastoma multiforme (GBM), Head and neck squamous cell carcinoma (HNSC), Kidney chromophobe (KICH), Kidney renal clear cell carcinoma (KIRC), Kidney renal papillary cell carcinoma (KIRP), Brain lower grade glioma (LGG), Liver hepatocellular carcinoma (LIHC), Lung adenocarcinoma (LUAD), Lung squamous cell carcinoma (LUSC), Mesothelioma (MESO), Ovarian serous cystadenocarcinoma (OV), Pancreatic adenocarcinoma (PAAD), Pheochromocytoma and paraganglioma (PCPG), Prostate adenocarcinoma (PRAD), Rectum adenocarcinoma (READ), Sarcoma (SARC), Skin cutaneous melanoma (SKCM), Stomach adenocarcinoma (STAD), Testicular germ cell tumors (TGCT), Thyroid carcinoma (THCA), Thymoma (THYM), Uterine corpus endometrial carcinoma (UCEC), Uterine carcinosarcoma (UCS), and Uveal melanoma (UVM). *, **, and *** indicate *p* < 0.05, *p* < 0.01, and *p* < 0.001, respectively.

### Association of METTL6 expression with clinical parameters in patients with GC

The clinicopathological characteristics of patients with gastric cancer in the TCGA cohort are summarized in Table 1. A total of 375 patients were included for sex analysis, 371 for age, 367 for pathological T stage, 357 for N stage, 355 for M stage, 352 for pathological stage, 364 for histological grade, 329 for residual tumor status, and 375 for overall survival analysis. Males accounted for 64.27% of the cohort, and 55.80% of patients were older than 65 years.

**Table 1:** Clinicopathological characteristics of patients with gastric cancer in the TCGA cohort.

Characteristics	Subgroups	Number of patients (N)	Percentage (%)
Sex	Total	375	100.00%
	Female	134	35.73%
	Male	241	64.27%
Age (years)	Total	371	100.00%
	≤ 65	164	44.20%
	>65	207	55.80%
Pathologic T stage	Total	367	100%
	T1	19	5.18%
	T2	80	21.80%
	T3	168	45.78%
	T4	100	27.25%
Pathologic N stage	Total	357	100%
	N0	111	31.09%
	N1	97	27.17%
	N2	75	21.01%
	N3	74	20.73%
Pathologic M stage	Total	355	100%
	M0	330	92.96%
	M1	25	7.04%
Pathological stage	Total	352	100.00%
	Stage I + II	164	46.59%
	Stage III + IV	188	53.41%
Histologic grade	Total	364	100.00%
	G1	10	2.75%
	G2	137	37.64%
	G3	217	59.62%
Residual tumor (R)	Total	329	100.00%
	R0	298	90.58%
	R1	15	4.56%
	R2	16	4.86%
OS event	Total	375	100.00%
	Alive	228	60.80%
	Dead	147	39.20%

Regarding tumor stage distribution, most patients presented with advanced disease. In the pathological T stage, T3 (45.78%) and T4 (27.25%) were the most frequent categories. For lymph node status, N0–N1 cases accounted for 58.26% of patients, while N2–N3 accounted for 41.74%. Distant metastasis analysis showed that the majority of patients were classified as M0 (92.96%), with M1 disease accounting for 7.04%.

According to overall pathological stage, stage III–IV patients accounted for 53.41% of the cohort, whereas stage I–II accounted for 46.59%. Histological grading showed that G3 tumors were the most common subtype (59.62%), followed by G2 (37.64%) and G1 (2.75%). Residual tumor analysis indicated that R0 resection was achieved in 90.58% of cases, while R1 and R2 accounted for 4.56% and 4.86%, respectively.

To further investigate the relationship between METTL6 expression and clinical features, TCGA-STAD patients were stratified based on clinicopathological variables. As shown in Fig. 2, METTL6 expression was significantly associated with sex (female *vs.* male, Fig. 2A), T stage (T1–T4, Fig. 2C), N stage (N0–N3, Fig. 2D), M stage (M0, M1, Fig. 2E), pathological stage (I–IV, Fig. 2F), histologic grade (G1–G3, Fig. 2G), residual tumor status (R0–R2, Fig. 2H), and overall survival status (alive *vs.* dead, Fig. 2I). These results suggest that METTL6 expression is closely correlated with disease progression and tumor aggressiveness in gastric cancer.

**Figure 2:**
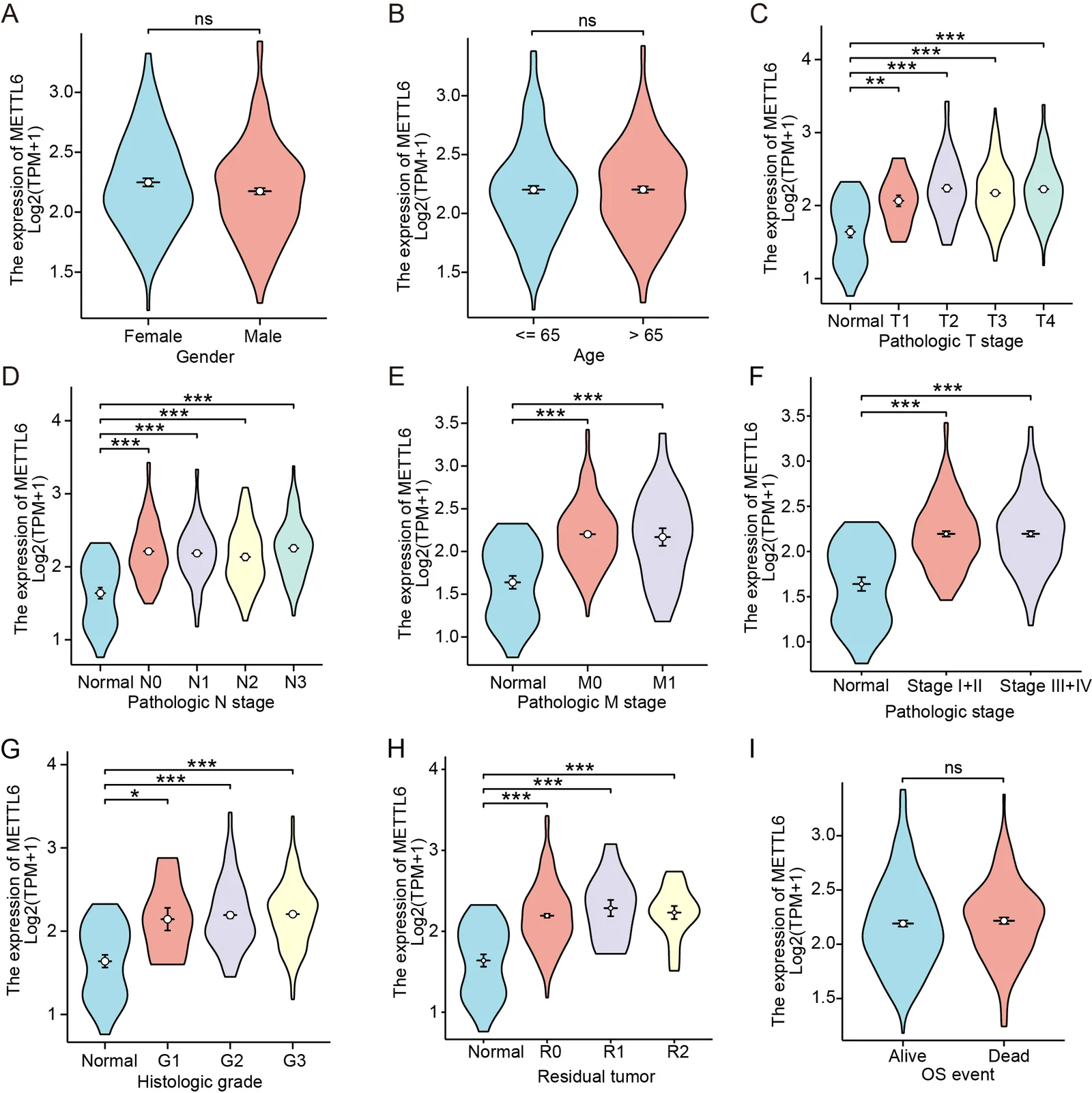
Association between methyltransferase-like 6 (METTL6) expression and clinicopathological characteristics in gastric cancer (GC) based on The Cancer Genome Atlas (TCGA) data. (A) Gender; (B) Age; (C) pathologic T stage; (D) pathologic N stage; (E) pathologic M stage; (F) pathologic stage; (G) histologic grade; (H) residual tumor; (I) OS event. Statistical significance was determined using the Wilcoxon rank-sum test for two-group comparisons (A, B, and I), One-way ANOVA for panel C, and the Kruskal–Wallis test for multi-group ordinal comparisons (D–H). Data are displayed as box-and-whisker plots. Asterisks (*, **, and ***) indicate *p* < 0.05, *p* < 0.01, and *p* < 0.001, respectively.

### Prognostic and diagnostic significance of METTL6 expression in GC

To assess the prognostic relevance of METTL6 expression in patients with GC, STAD cases were stratified into high- and low-expression groups as described above. Kaplan–Meier Plotter analysis demonstrated that elevated METTL6 expression was significantly associated with poorer outcomes in terms of OS, FP, and PPS (Figs. 3A–3C). These findings were further validated using an independent public dataset (Figs. 3D–3H). Subgroup analyses based on clinicopathological characteristics revealed that high METTL6 expression was strongly correlated with worse prognosis in patients with pathological N2 stage, pathological M1 stage, and those who achieved complete remission as the primary therapy outcome (Figs. 3I–3K). Survival events (OS, FP, PPS) were ascertained from documented dates of diagnosis, last follow-up, and death or progression events in TCGA-STAD annotations. Hazard ratios (HRs) and 95% confidence intervals (CIs) were calculated using univariate Cox proportional hazards models implemented through XIANTAO survival modules.

**Figure 3:**
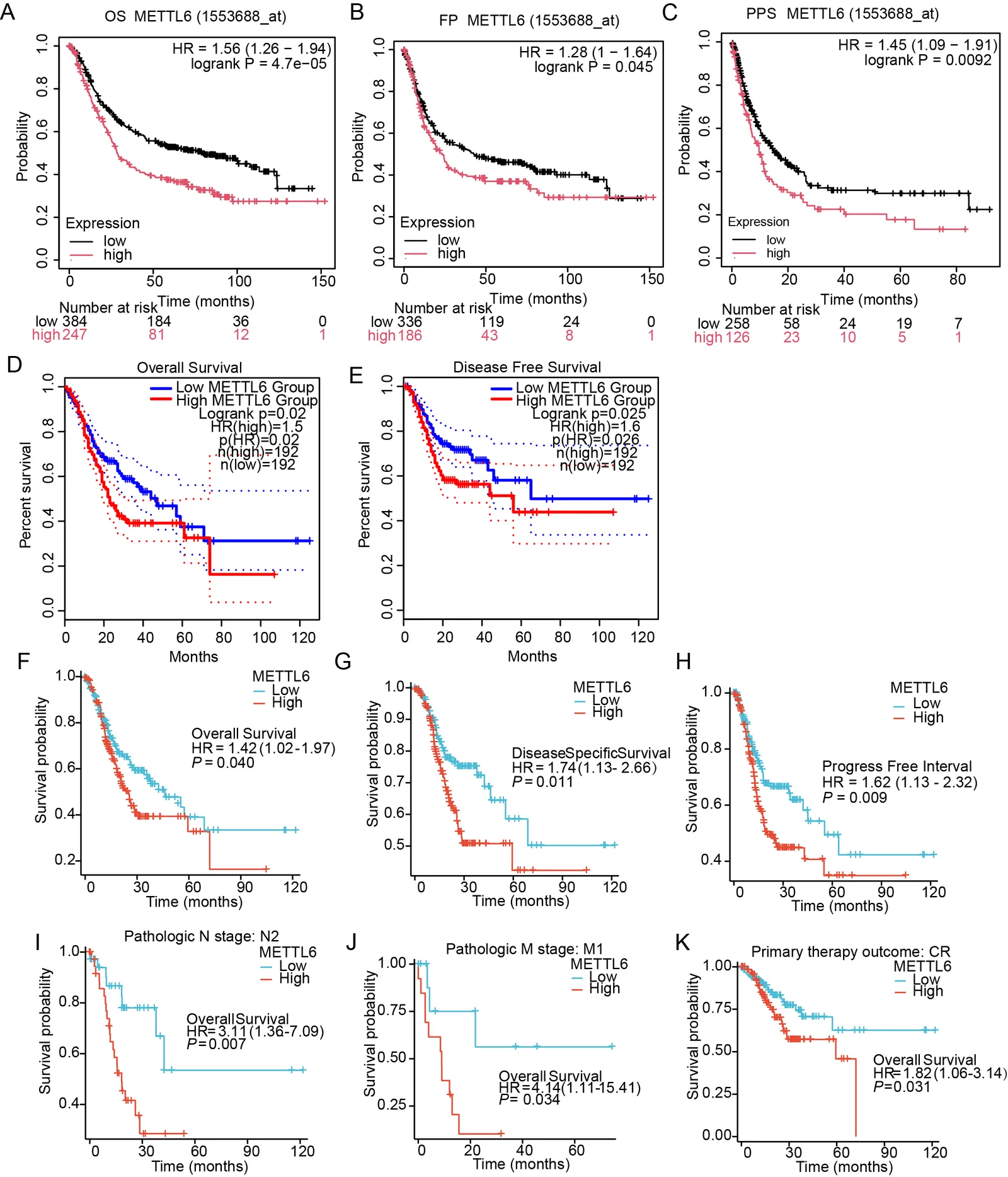
High expression of METTL6 predicted poor outcomes. (A-C) Kaplan–Meier survival curves were used to express OS, FP, and PPS. (D–E) OS and DFS were expressed based on the GEPIA database. (F–K) Prognostic subgroup analyses of GC individuals with varying clinical and pathological characteristics. Patients were stratified into high- and low-expression groups based on a median expression split (0–50% *vs.* 50–100%). Statistical significance and hazard ratios (HRs) with 95% confidence intervals (CIs) were evaluated using the Log-rank (Mantel-Cox) test and univariate Cox proportional hazards regression models.

### Analysis of METTL6 association and genetic alterations

To investigate the protein–protein interaction network and elucidate the associations between METTL6 and other proteins in GC, the STRING database was first used to construct the interaction network (Fig. 4A). In addition, the GEPIA2 database was employed to identify genes associated with METTL6 expression. As shown in Figs. 4B–4D, METTL6 exhibited strong positive correlations with the three most highly associated genes: CAPN7 (*R* = 0.64), EAF1 (*R* = 0.68), and MKRN2 (*R* = 0.67). Heatmap analysis further demonstrated significant associations between METTL6 and these three genes across multiple cancer types (Fig. 4E). Moreover, intersection analysis of the two predicted gene sets identified three common genes: CWF19L1, EAF1, and NME6 (Fig. 4F).

**Figure 4:**
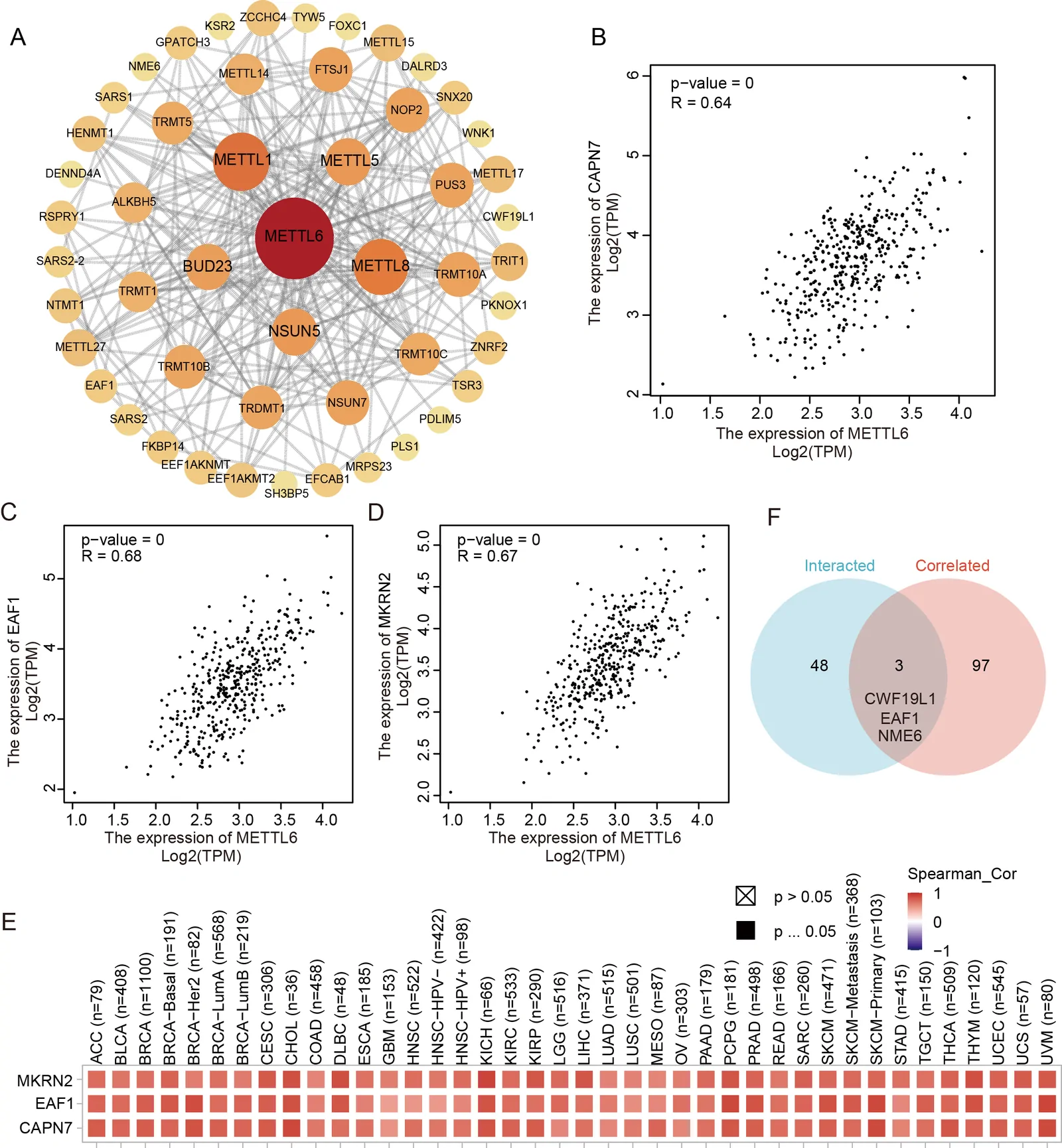
Interaction and correlation analysis of methyltransferase-like 6 (METTL6)-binding proteins. (A) Protein–protein interaction network of METTL6-binding proteins generated using STRING. (B–D) Correlations between METTL6 expression and CAPN7, EAF1, and MKRN2 based on the top 100 METTL6-related genes from The Cancer Genome Atlas (TCGA), analyzed using GEPIA2. (E) Heatmap showing gene correlations across multiple tumor types. (F) Intersection analysis of METTL6-associated and interacting genes. Correlation coefficients (R) and statistical *p*-values were calculated using Pearson or Spearman correlation tests within the respective web servers.

Functional enrichment analysis was performed to predict the biological roles of the top 100 genes associated with METTL6 (Fig. 5). Gene Ontology enrichment analysis revealed that the most significantly enriched terms included “RNA modification,” “methylation,” “RNA methylation,” and “RNA methyltransferase activity.” Kyoto Encyclopedia of Genes and Genomes pathway analysis indicated that these genes were predominantly involved in “Nucleotide metabolism,” “Aminoacyl-transfer RNA (tRNA) biosynthesis,” and “Pyrimidine metabolism.”

**Figure 5:**
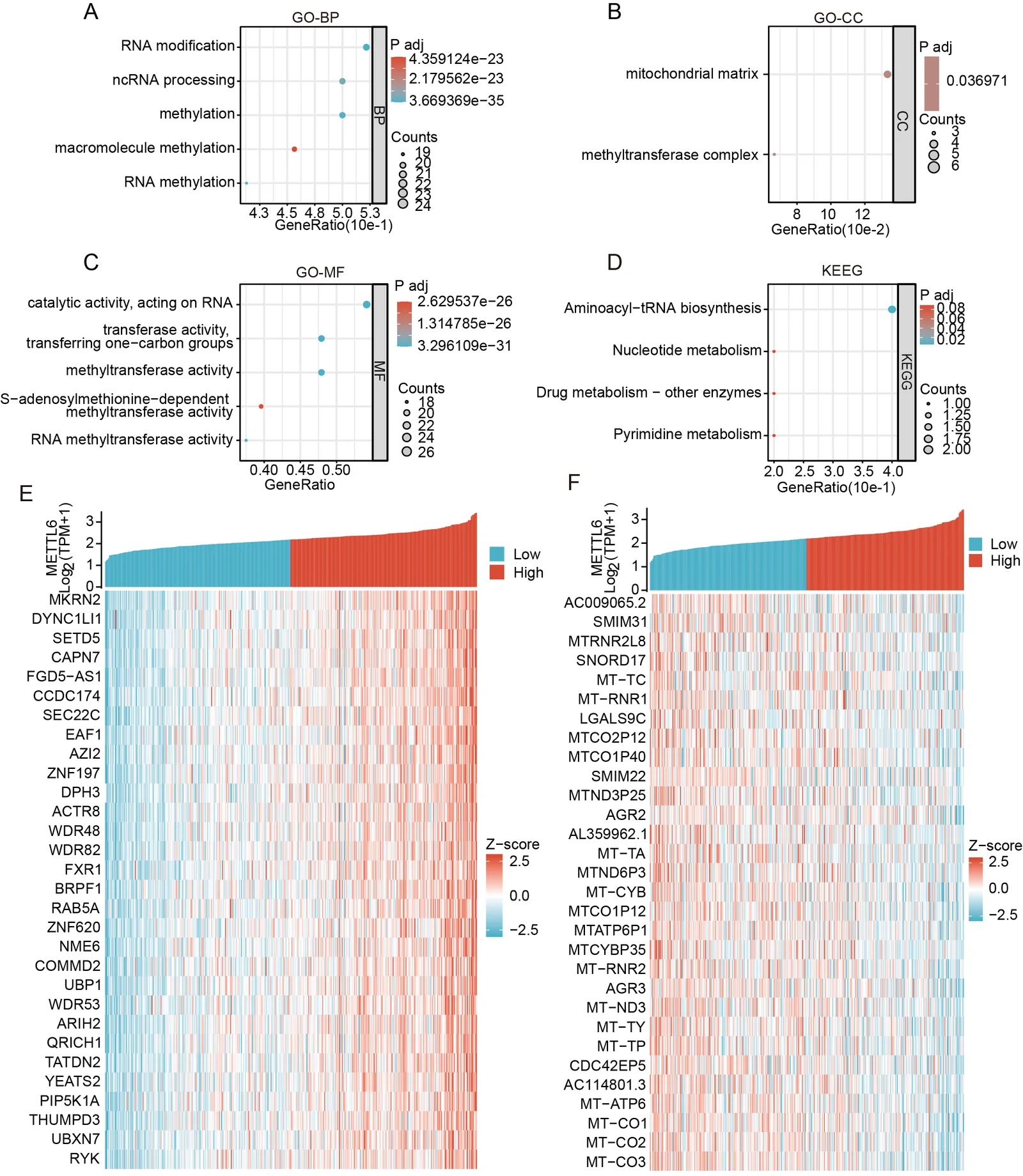
Functional enrichment and gene correlation analysis of METTL6 in GC. (A–D) The 100 METTL6-interacting and correlated genes were utilized for GO and KEGG enrichment analyses. (E–F) Heat maps showing the top 30 genes positively and negatively correlated with METTL6 in GC. Statistical significance for enrichment terms was adjusted using the Benjamini–Hochberg false discovery rate (FDR) method. Heatmap color scales reflect the strength of the correlation coefficients.

### Association between METTL6 expression and infiltrating immune cells

The TIMER2.0 database was used to evaluate the relationship between METTL6 expression and immune cell infiltration in GC. As shown in Figs. 6A–6F, METTL6 expression was positively correlated with CD4+ T cells, CD8+ T cells, dendritic cells, neutrophils, and macrophages, whereas no significant association was observed with B cells in GC. Subsequently, the associations between METTL6 expression and 24 immune cell subtypes were examined. The results demonstrated increased levels of T helper cells and central memory T cells (Tcm) in patients with elevated METTL6 expression, while plasmacytoid dendritic cells and T helper 17 cells were decreased (Fig. 6G). In addition, METTL6 expression showed significant positive correlations with 9 of the 24 immune cell subtypes (Fig. 6H).

**Figure 6:**
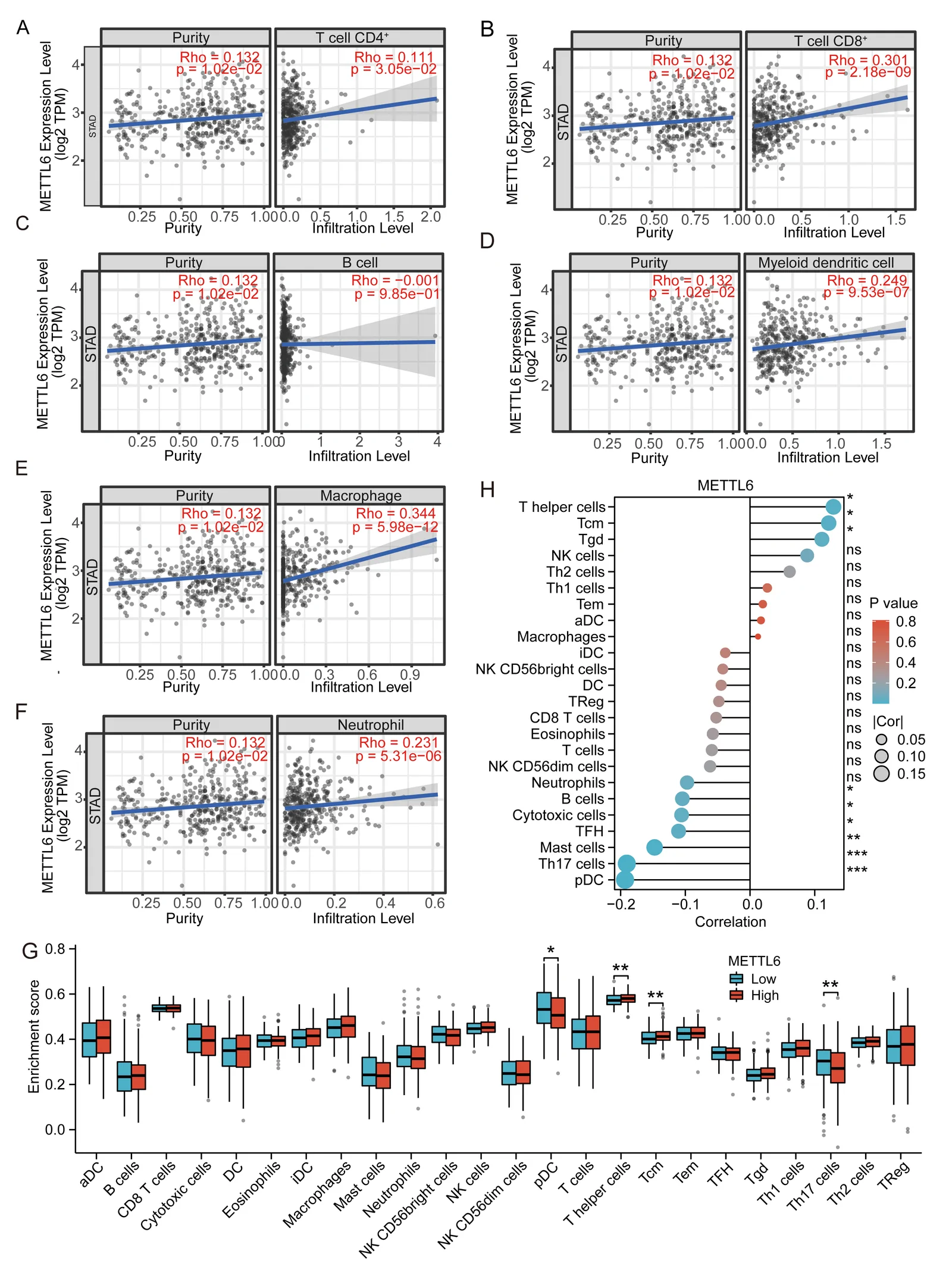
Correlation of METTL6 expression with immune infiltration and subtype distribution in GC. (A–F) This correlation was analyzed using the TIMER database. (G) Comparison of 24 immune cell subtype levels between high and low METTL6 expression groups. (H) Spearman’s analysis of METTL6 expression and immune infiltration levels visualized as a correlation coefficient distribution. Asterisks (*, **, and ***) indicate *p* < 0.05, *p* < 0.01, and *p* < 0.001, respectively.

We further analyzed the relationship between METTL6 expression and the tumor microenvironment (TME) across multiple tumor types in TCGA. The results revealed a strong and significant correlation between METTL6 expression and estimated infiltration levels of cancer-associated fibroblasts in the majority of cancers (Fig. 7A). Figure 7B and Fig. S1 present scatterplot data generated using a highly correlated algorithm. Finally, we investigated the prognostic impact of METTL6 expression in conjunction with immune cell infiltration in patients with GC. The results indicated that under conditions of enriched infiltration of CD4+ and CD8+ memory T cells, eosinophils, natural killer T cells, regulatory T cells, and T helper two cells, together with reduced infiltration of basophils, B cells, macrophages, mesenchymal stem cells, and T helper 1 cells, high METTL6 expression predicted a poor prognosis in GC (Fig. 8 and Table S2).

**Figure 7:**
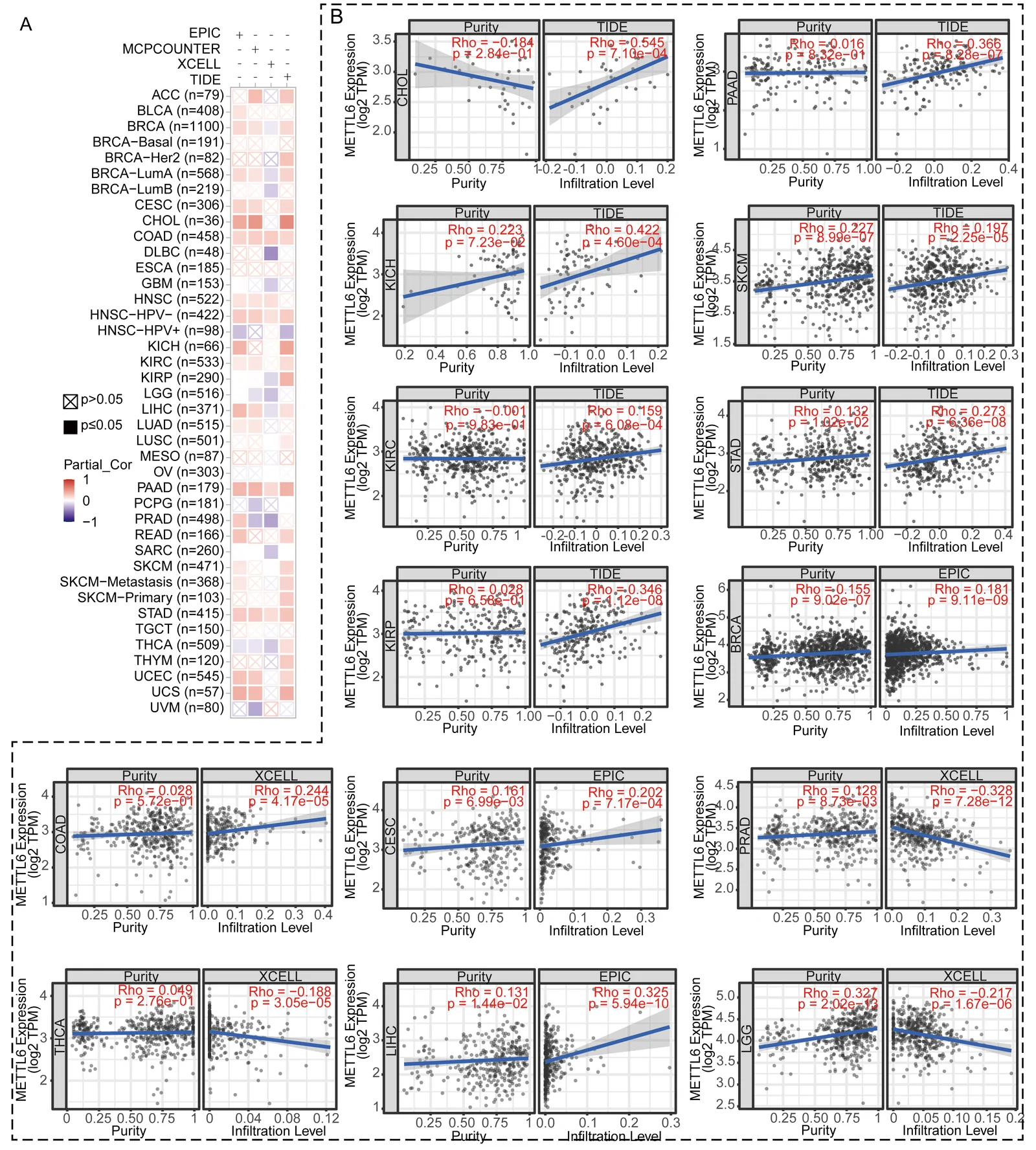
Association between methyltransferase-like 6 (METTL6) expression and immune cell infiltration across The Cancer Genome Atlas (TCGA) cancer types. (A) Heatmap showing correlations between METTL6 expression and infiltration levels of cancer-associated fibroblasts (CAFs) and other immune cells across multiple cancer types using the EPIC, MCPCOUNTER, XCELL, and TIDE algorithms. (B) Scatter plots showing associations between METTL6 expression and CAF infiltration across multiple tumor types, including cholangiocarcinoma (CHOL), pancreatic adenocarcinoma (PAAD), kidney chromophobe (KICH), skin cutaneous melanoma (SKCM), kidney renal clear cell carcinoma (KIRC), stomach adenocarcinoma (STAD), kidney renal papillary cell carcinoma (KIRP), breast invasive carcinoma (BRCA), colon adenocarcinoma (COAD), cervical squamous cell carcinoma and endocervical adenocarcinoma (CESC), prostate adenocarcinoma (PRAD), thyroid carcinoma (THCA), liver hepatocellular carcinoma (LIHC), and low-grade glioma (LGG), based on the algorithm demonstrating the strongest correlation. Statistical significance and correlation metrics were determined by the standard procedures inherent to each corresponding deconvolution algorithm.

**Figure 8:**
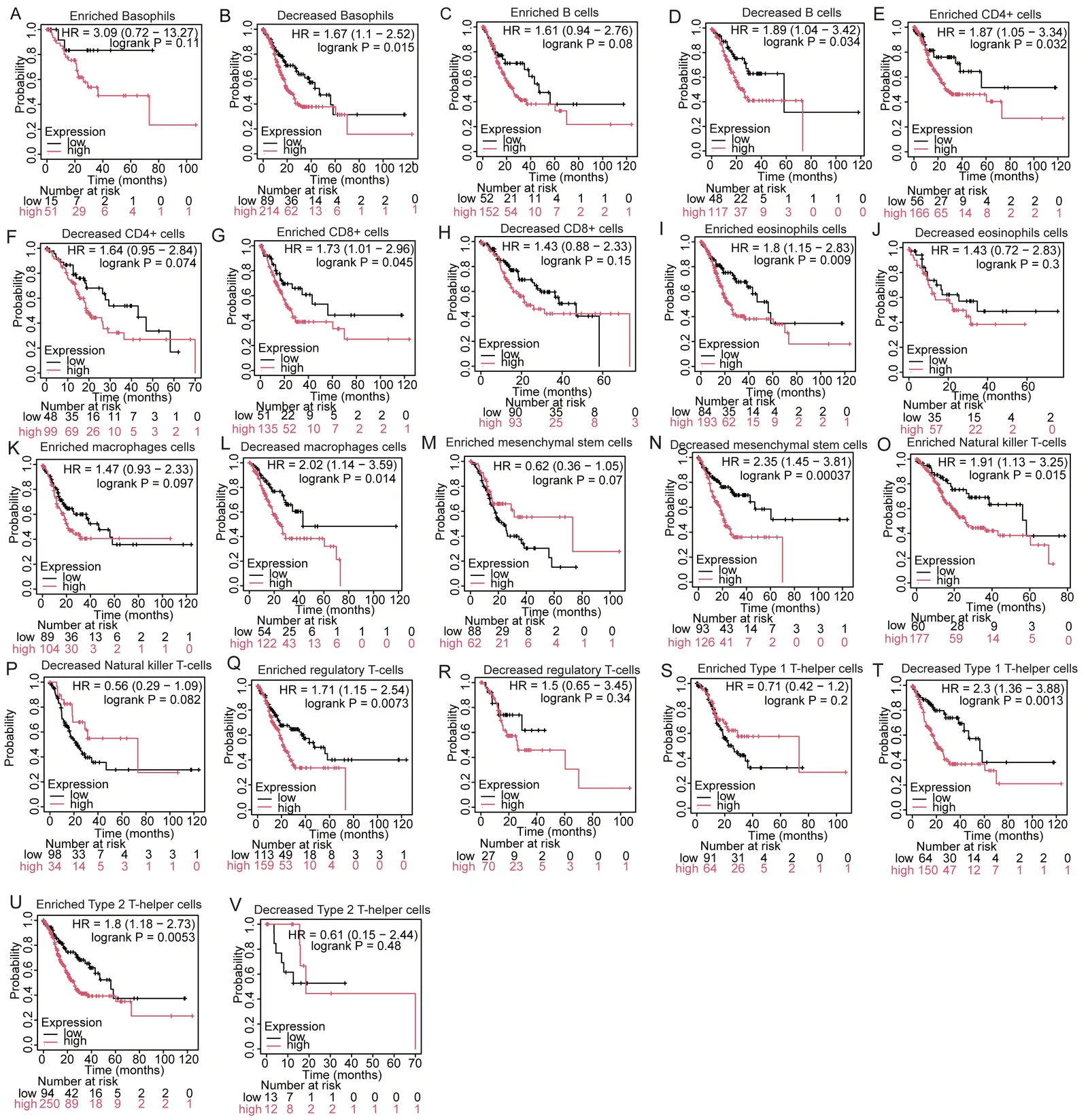
Predictive value of methyltransferase-like 6 (METTL6) expression in patients with gastric cancer (GC) across various immune cell subsets. Kaplan–Meier survival curves showing the prognostic impact of METTL6 expression combined with enriched or decreased infiltration of immune cell subsets, including basophils (A and B), B cells (C and D), CD4+ T cells (E and F), CD8+ T cells (G and H), eosinophils (I and J), macrophages (K and L), mesenchymal stem cells (M and N), natural killer T cells (O and P), regulatory T cells (Q and R), T helper 1 cells (S and T), and T helper 2 cells (U and V). Prognostic differences between groups were evaluated using the Log-rank (Mantel-Cox) test.

**Figure 9:**
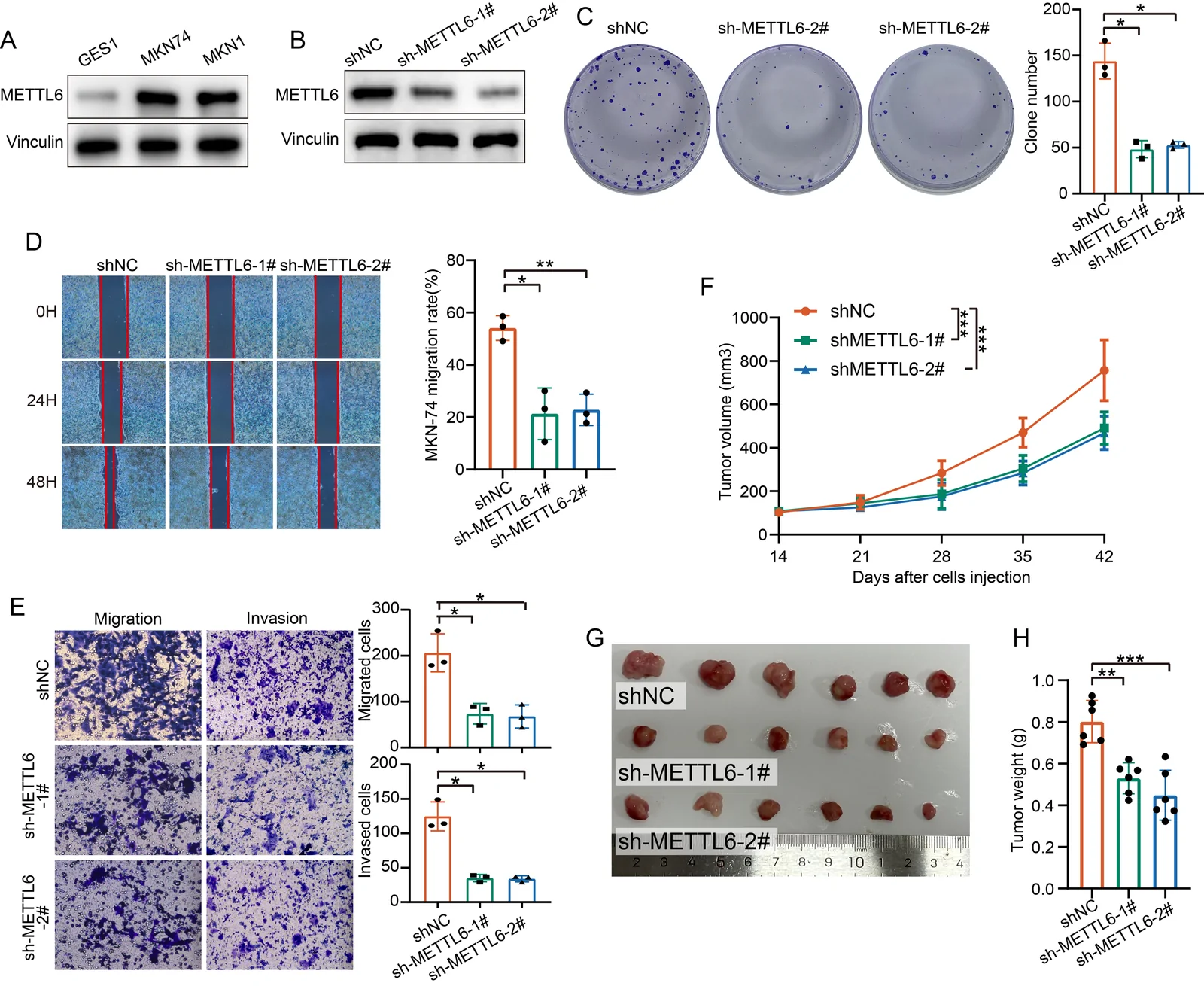
Methyltransferase-like 6 (METTL6) knockdown inhibits gastric cancer (GC) progression *in vitro* and *in vivo*. (A) METTL6 protein expression in GC cell lines quantified relative to Vinculin, with relative densitometric values indicated beneath the bands. (B) Knockdown efficiency of METTL6 short hairpin RNA (shRNA) in MKN-74 and MKN-1 cells, with relative densitometric values indicated beneath the bands. (C–E) Representative images and corresponding quantitative analyses of colony formation (C), wound healing (D), migration and invasion assays (E) in control and shMETTL6 groups. (F) Tumor volume was measured from tumor implantation until sacrifice. (G) Representative tumor images after excision. (H) Tumor weight was measured after sacrifice. All *in vitro* experiments were independently repeated at least three times (*n* = 3 biological replicates per experiment), and results are presented as mean ± SD. Statistical evaluations were performed using Student’s *t*-test or one-way ANOVA followed by Tukey’s *post-hoc* test. Asterisks (*, **, and ***) indicate *p* < 0.05, *p* < 0.01, and *p* < 0.001, respectively.

### Downregulation of METTL6 decreased the aggressiveness of GC cells *in vitro*

To further validate the involvement of METTL6 in GC, a series of functional experiments was performed. A marked increase in METTL6 expression was first observed in two GC cell lines, MKN-74 and MKN-1, compared with normal gastric epithelial cells (Fig. 9A). METTL6 expression was subsequently suppressed in both MKN-74 and MKN-1 cells using lentiviral-mediated knockdown (Fig. 9B). The colony formation assay demonstrated that METTL6 knockdown significantly reduced colony formation, indicating impaired proliferative capacity of GC cells (Fig. 9C). In addition, wound healing and Transwell assays showed that METTL6 knockdown markedly decreased the migratory and invasive abilities of these cells, confirming reduced metastatic potential (Figs. 9D and 9E).

### Knockdown of METTL6 suppresses GC growth *in vivo*

To further examine the effects of METTL6 suppression *in vivo*, xenograft tumor models were established in nude mice. Compared with the control group, METTL6 knockdown significantly inhibited tumor growth on days 28, 35, and 42 (Fig. 9F and Table S1). On the final day of observation, tumor volume analysis showed that METTL6 suppression by shRNA-1 and shRNA-2 reached 42.24 ± 11.91% (*p* < 0.001) and 44.78 ± 14.88% (*p* < 0.001), respectively. At the study endpoint, mice were euthanized, and tumors were excised, photographed, and weighed. As shown in Figs. 9G and 9H, METTL6 knockdown resulted in significant reductions in both tumor size and tumor weight. Tumor weight analysis revealed that METTL6 inhibition by shRNA-1 and shRNA-2 was 34.49 ± 12.41% (*P* = 0.002) and 45.91 ± 13.48% (*p* < 0.001), respectively. Collectively, these results demonstrate that METTL6 knockdown suppresses GC progression in a xenograft model, providing proof-of-concept evidence for the functional importance of METTL6 and supporting its potential as a therapeutic target in GC.

## Discussion

In this study, analysis of the TCGA database revealed significantly elevated METTL6 expression in STAD tumor tissues compared with paracancerous tissues. Further assessment of protein expression levels confirmed this upregulation, supporting the potential of METTL6 as a promising biomarker and warranting further investigation of its therapeutic relevance in GC.

Prognostic biomarkers provide critical insight into tumor progression and patient outcomes and play an essential role in personalized and precision medicine by helping to avoid inappropriate treatment strategies. We identified a strong association between METTL6 expression and both tumor TNM stage and histologic grade, indicating an important role for METTL6 in the infiltrative growth of GC. Further analyses of OS, FP, and PPS across multiple patient subgroups consistently demonstrated that elevated METTL6 expression was associated with poorer survival. Collectively, these findings establish that METTL6 is a reliable prognostic predictor in patients with GC.

RNA modification is a major regulatory mechanism of gene expression, involving chemical alterations of RNA bases and ribose moieties. To date, more than 170 distinct RNA modifications have been identified across different RNA species in both prokaryotic and eukaryotic organisms. Among these, RNA methylation accounts for over 60% of all RNA modifications and plays a critical role in post-transcriptional gene regulation (Barbieri & Kouzarides, 2020; Cui et al., 2022; Li et al., 2022; Xue et al., 2022). The principal forms of RNA methylation, including N1-methyladenosine (m^1^A), N6-methyladenosine (m^6^A), 5-methylcytosine (m^5^C), N7-methylguanosine (m^7^G), and 3-methylcytidine (m^3^C), underscore the extensive prevalence and biological importance of this modification class (Chen et al., 2023b; Han & Xu, 2023; Li et al., 2024). Various RNA methylations are mediated by a diverse family of METTLs, comprising both characterized and predicted methyltransferases (Ignatova et al., 2019). Numerous METTL family members have been implicated in the development and progression of multiple cancer types (Deng et al., 2018). Recent work has identified METTL6 as a bona fide tRNA methyltransferase that catalyzes the formation of 3-methylcytidine at position C32 in specific serine tRNA isoacceptors (Ignatova et al., 2020). Although our bioinformatic findings, together with the established biochemical function of METTL6 as a tRNA m^3^C methyltransferase, suggest that its catalytic activity contributes to the observed oncogenic phenotypes, direct experimental confirmation of this mechanism, such as through the use of catalytically inactive mutants, was not performed in the present study and represents an important direction for future investigation.

Comprehensive investigation of TME has yielded important insights into the molecular classification of cancers (Becht et al., 2016; Fridman et al., 2012; Jiang et al., 2018a; Jiang et al., 2018b; Mahajan et al., 2018; Zeng et al., 2018). Quantitative characterization of distinct cellular subpopulations within TME has enabled the identification of multiple biomarkers associated with patient prognosis and therapeutic response, including responsiveness to chemotherapy and immunotherapy (Jiang et al., 2018a; Mahajan et al., 2018). In GC, prior studies have demonstrated that TME exerts a substantial influence on clinical outcome and that dysregulation of TME contributes to treatment resistance (Jiang et al., 2018a). Notably, patients with tumors enriched in cytotoxic CD8+ T lymphocytes exhibit prolonged survival, whereas elevated neutrophil infiltration is associated with poor prognosis (Jiang et al., 2018a; Jiang et al., 2018b). In the present study, patients with high METTL6 expression displayed increased CD8+ T lymphocyte infiltration. This phenomenon may reflect the influence of additional immunoregulatory components within the TME, including macrophages, dendritic cells, and fibroblasts, which can modulate CD8+ T cell activity. Moreover, METTL6 expression exhibited strong correlations with the abundance of four immune cells: T helper 17, plasmacytoid dendritic cells, Tcm, and T helper cells. Collectively, these findings indicate that METTL6 expression is closely associated with immune cell infiltration in GC. However, whether METTL6 directly regulates immune cell recruitment or function and thereby influences patient prognosis remains speculative and requires further experimental validation.

Given the potential link between elevated METTL6 expression and tumorigenesis, downregulation of METTL6 may represent a potential strategy for inhibiting tumor progression. In the present study, METTL6 knockdown significantly suppressed GC cell proliferation, migration, and invasion *in vitro* and markedly inhibited tumor growth in a murine xenograft model. These results provide compelling evidence that METTL6 contributes to GC progression. Nevertheless, the *in vivo* xenograft experiments were limited by a relatively modest sample size (*n* = 6 per group). Although this sample size was determined based on pilot data and is commonly employed in xenograft studies, validation in larger cohorts will be necessary to confirm the robustness of these findings. Moreover, the current *in vivo* experiments relied on shRNA-mediated gene silencing, which serves as a proof-of-concept approach but does not constitute a clinically applicable therapeutic strategy. Future studies should focus on the development of pharmacological METTL6 inhibitors and rigorous evaluation of their specificity, safety, and therapeutic efficacy.

Several limitations of this study should be acknowledged. First, although we demonstrated that METTL6 is significantly overexpressed in gastric cancer and that its knockdown suppresses tumor cell proliferation, migration, invasion, and *in vivo* tumor growth, the present study does not establish whether these phenotypic effects are directly mediated by the catalytic tRNA methyltransferase activity of METTL6. METTL6 has been reported to function as a tRNA m^3^C methyltransferase; however, we did not perform rescue experiments using catalytically inactive mutants or enzymatic activity–deficient constructs. Therefore, it remains unresolved whether the observed oncogenic phenotypes are dependent on its methyltransferase activity or whether they may also involve non-catalytic functions, such as protein–protein interactions or structural/scaffolding roles. Second, although lentiviral shRNA-mediated knockdown provides functional evidence supporting a role for METTL6 in gastric cancer progression, potential off-target effects of RNA interference cannot be fully excluded. Future studies employing CRISPR/Cas9-mediated knockout and genetic rescue approaches would further strengthen causal inference. Third, the mechanistic interpretation of bioinformatic analyses based on TCGA and other public databases should be made with caution, as these analyses rely on retrospective datasets and computational deconvolution algorithms that may introduce inherent bias. Finally, the lack of validation in large independent clinical cohorts limits the translational generalizability of the findings. Additional prospective studies using well-characterized patient samples will be necessary to confirm the diagnostic and prognostic utility of METTL6 in gastric cancer.

## Conclusion

In conclusion, this study demonstrates that METTL6 is a key regulator of GC progression. By integrating large-scale bioinformatic analyses with experimental validation, we show that METTL6 is significantly overexpressed in GC and closely associated with adverse clinicopathological features, immune cell infiltration, and poor patient outcomes. Functional experiments further confirm that METTL6 promotes tumor cell proliferation, migration, invasion, and *in vivo* tumor growth. Collectively, these findings support METTL6 as a promising prognostic biomarker and potential therapeutic target in GC. Although further mechanistic and translational studies are required, this work provides a strong foundation for future efforts aimed at improving clinical management and therapeutic strategies for patients with GC.

##  Supplemental Information

10.7717/peerj.21763/supp-1 Supplemental Information 1 Association between immune cell infiltration and methyltransferase-like 6 (METTL6) expression across The Cancer Genome Atlas (TCGA) cancer types

10.7717/peerj.21763/supp-2 Supplemental Information 2 The forest plot summarizing the predictive value on METTL6 expression in patients with GC according to different immune cell subsets

10.7717/peerj.21763/supp-3 Supplemental Information 3 Tumor volumes in the subcutaneous xenograft model

10.7717/peerj.21763/supp-4 Supplemental Information 4 Overview of statistical methods and R packages utilized for bioinformatic analyses

10.7717/peerj.21763/supp-5 Supplemental Information 5 Tumor photograph

10.7717/peerj.21763/supp-6 Supplemental Information 6 Raw data

10.7717/peerj.21763/supp-7 Supplemental Information 7 Western blots

10.7717/peerj.21763/supp-8 Supplemental Information 8 Statistical analysis of METTL6

10.7717/peerj.21763/supp-9 Supplemental Information 9 ARRIVE guidelines

## Additional Information and Declarations

### Data Availability

The following information was supplied regarding data availability: The data is available in the Supplemental Files. The data is also available at Figshare: Wang (2026). Original data of METTL6. figshare. Dataset. https://doi.org/10.6084/m9.figshare.32726700.v2. XIANTAO and GEPIA2 were used as analysis interfaces rather than as primary data repositories. The underlying public datasets used in these analyses were derived from TCGA and GTEx. The TCGA Stomach Adenocarcinoma (TCGA-STAD) dataset is available at: https://portal.gdc.cancer.gov/projects/TCGA-STAD. TIMER/TIMER2.0 was also used as an analysis interface rather than as an independent primary dataset. TIMER2.0 performs immune-infiltration analyses using TCGA tumor data. Dataset name is TCGA Stomach Adenocarcinoma (TCGA-STAD) and the Project/accession: TCGA-STAD. The Genotype-Tissue Expression (GTEx) Project is available at: – dbGaP accession: phs000424 – https://dbgap.ncbi.nlm.nih.gov/beta/study/phs000424.v11.p2/#study. The gastric cancer analysis data is available through the specific cBioPortal dataset: – cBioPortal study ID: stad_tcga_pan_can_atlas_2018. – https://www.cbioportal.org/study/summary?id=stad_tcga_pan_can_atlas_2018. The gastric cancer Kaplan–Meier Plotter dataset integrates the following GEO gastric cancer cohorts used for survival analysis: – GSE14210: https://www.ncbi.nlm.nih.gov/geo/query/acc.cgi?acc=GSE14210. – GSE15459: https://www.ncbi.nlm.nih.gov/geo/query/acc.cgi?acc=GSE15459. - GSE22377: https://www.ncbi.nlm.nih.gov/geo/query/acc.cgi?acc=GSE22377. - GSE29272: https://www.ncbi.nlm.nih.gov/geo/query/acc.cgi?acc=GSE29272. - GSE51105: https://www.ncbi.nlm.nih.gov/geo/query/acc.cgi?acc=GSE51105. The STRING analysis was performed for human METTL6. Organism: Homo sapiens (NCBI taxonomy ID: 9606), STRING protein identifier: 9606.ENSP00000407613 – https://string-db.org/network/9606.ENSP00000407613.
