# Supplementary material for: Role of METTL6 in gastric cancer: diagnostic, prognostic, and functional implications
Source: PeerJ. 2026 Nov 2;14:e21763. doi: 10.7717/peerj.21763 (PMC13738193; doi:10.7717/peerj.21763)
Supplement: Supplementary file 1 [file peerj-14-21763-s001.pdf]

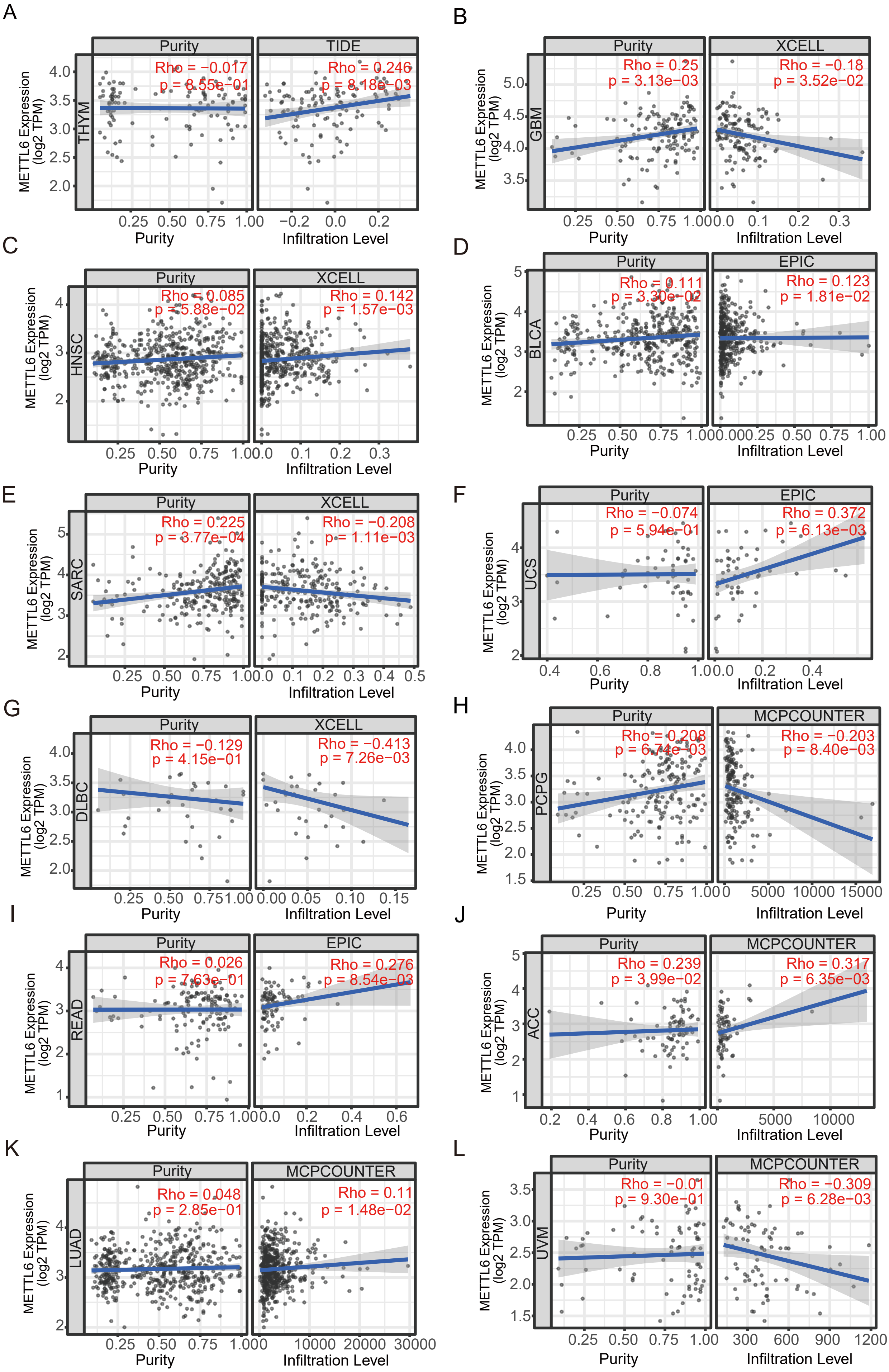
A
Purity
TIDE
THYM
Rho = −0.017
p = 8.55e−01
Rho = 0.246
p = 8.18e−03
METTL6 Expression (log2 TPM)
Infiltration Level
B
Purity
XCELL
GBM
Rho = 0.25
p = 3.13e−03
Rho = −0.18
p = 3.52e−02
C
Purity
XCELL
HNSC
Rho = 0.085
p = 5.88e−02
Rho = 0.142
p = 1.57e−03
D
Purity
EPIC
BLCA
Rho = 0.111
p = 3.30e−02
Rho = 0.123
p = 1.81e−02
E
Purity
XCELL
SARC
Rho = 0.225
p = 3.77e−04
Rho = −0.208
p = 1.11e−03
F
Purity
EPIC
UCS
Rho = −0.074
p = 5.94e−01
Rho = 0.372
p = 6.13e−03
G
Purity
XCELL
DLBC
Rho = −0.129
p = 4.15e−01
Rho = −0.413
p = 7.26e−03
H
Purity
MCPCOUNTER
PCPG
Rho = 0.208
p = 6.74e−03
Rho = −0.203
p = 8.40e−03
I
Purity
EPIC
READ
Rho = 0.026
p = 7.63e−01
Rho = 0.276
p = 8.54e−03
J
Purity
MCPCOUNTER
ACC
Rho = 0.239
p = 3.99e−02
Rho = 0.317
p = 6.35e−03
K
Purity
MCPCOUNTER
LUAD
Rho = 0.048
p = 2.85e−01
Rho = 0.11
p = 1.48e−02
L
Purity
MCPCOUNTER
UVM
Rho = −0.01
p = 9.30e−01
Rho = −0.309
p = 6.28e−03
