# Supplementary material for: Role of METTL6 in gastric cancer: diagnostic, prognostic, and functional implications
Source: PeerJ. 2026 Nov 2;14:e21763. doi: 10.7717/peerj.21763 (PMC13738193; doi:10.7717/peerj.21763)

Figure 1E

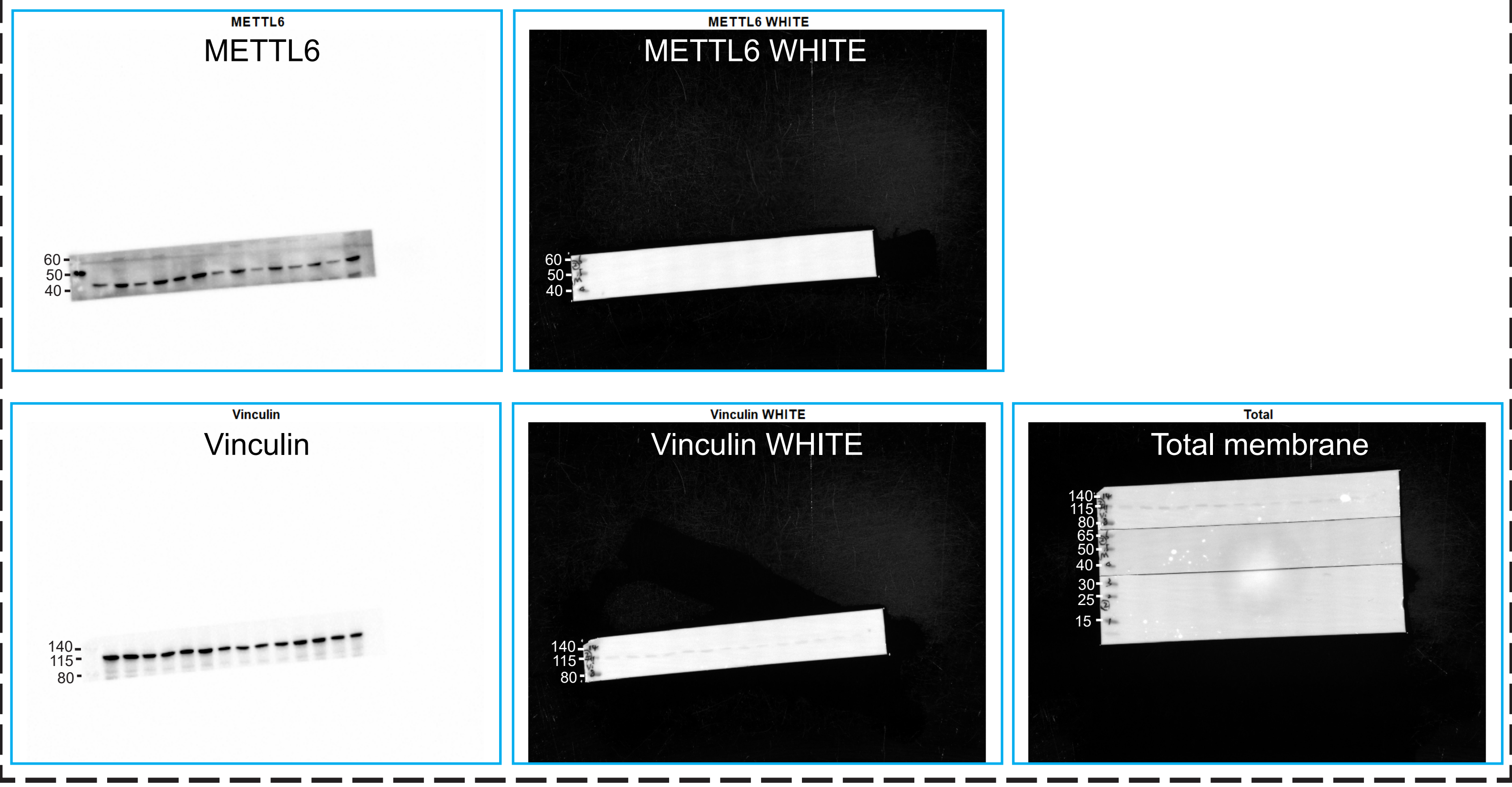

Figure 9A

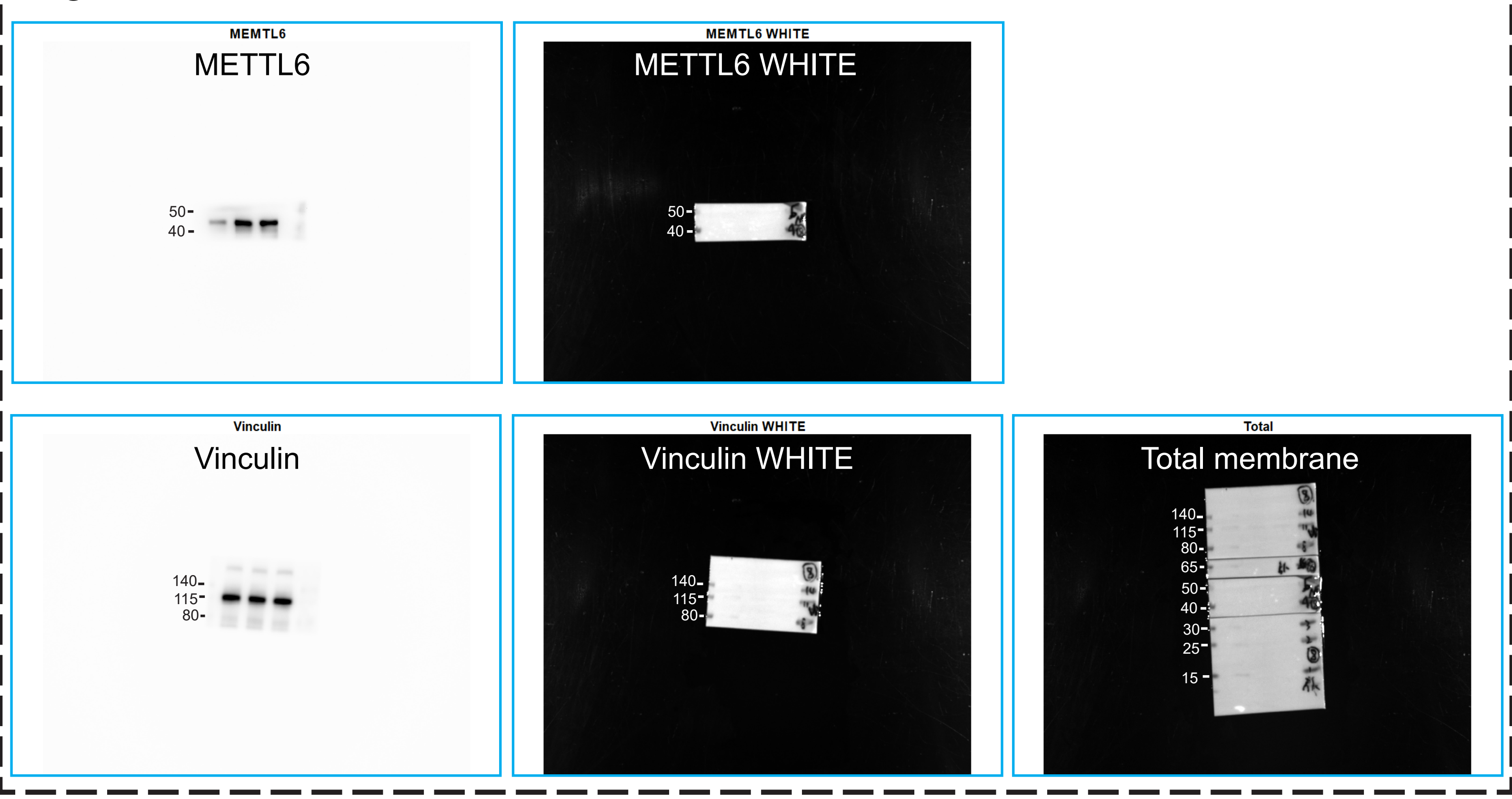

Figure 9B

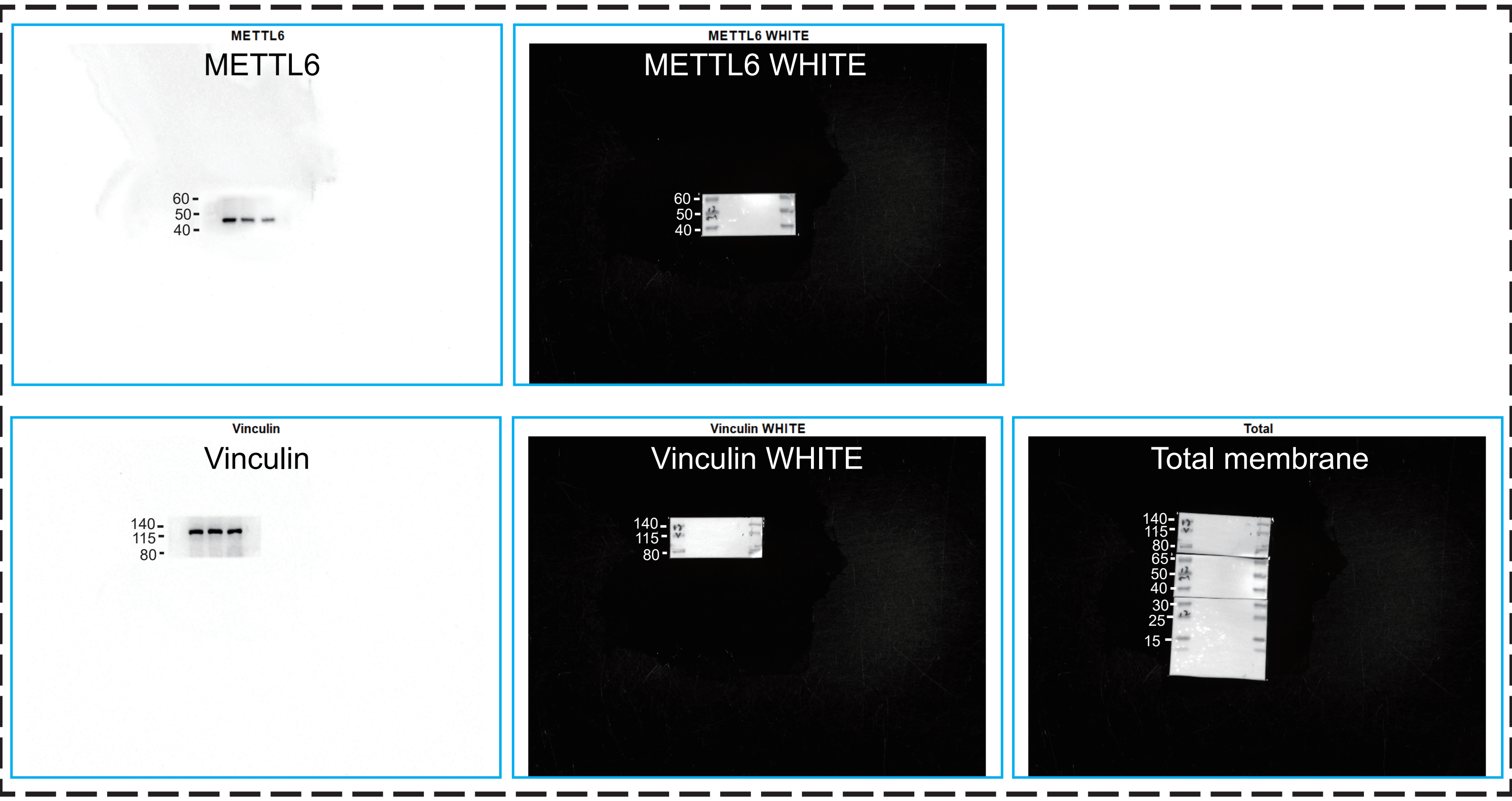
METTL6
METTL6
60
50
40
METTL6 WHITE
METTL6 WHITE
60
50
40
Vinculin
Vinculin
140
115
80
Vinculin WHITE
Vinculin WHITE
140
115
80
Total
Total membrane
140
115
80
65
50
40
30
25
15

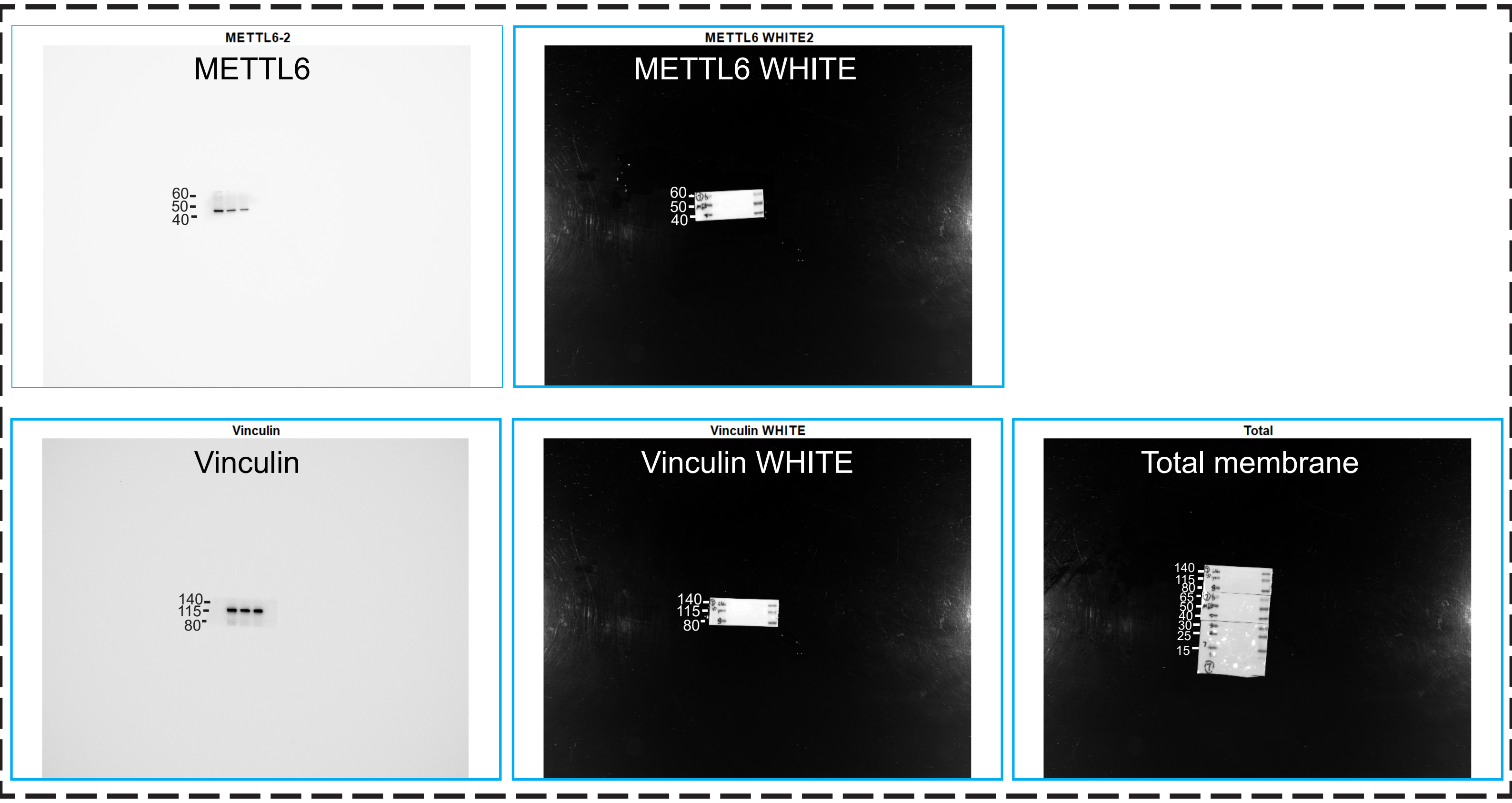
METTL6-2
METTL6
60
50
40
METTL6 WHITE2
METTL6 WHITE
60
50
40
Vinculin
Vinculin
140
115
80
Vinculin WHITE
Vinculin WHITE
140
115
80
Total
Total membrane
140
115
80
65
50
40
30
25
15

METTL6

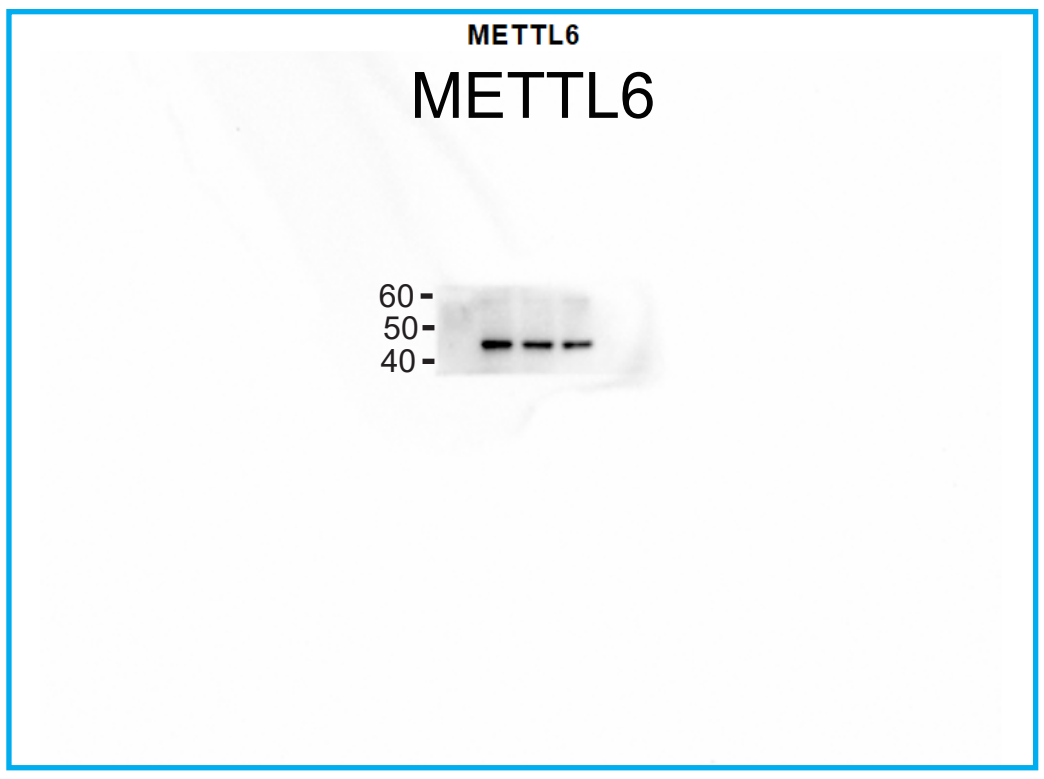

METTL6 WHITE

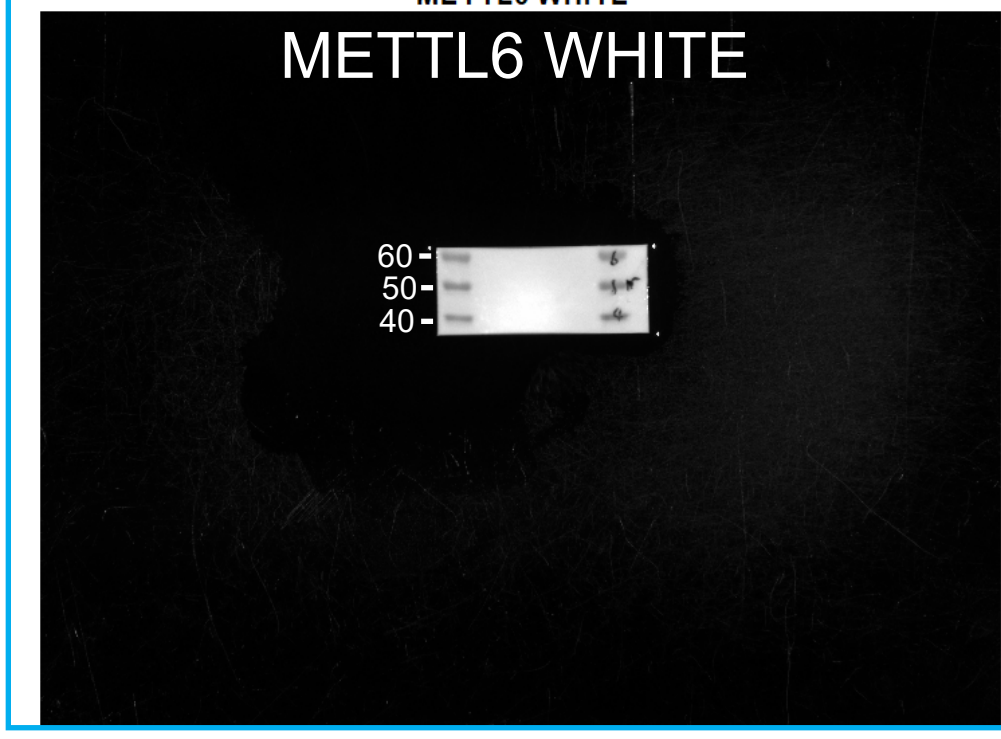

Vinculin

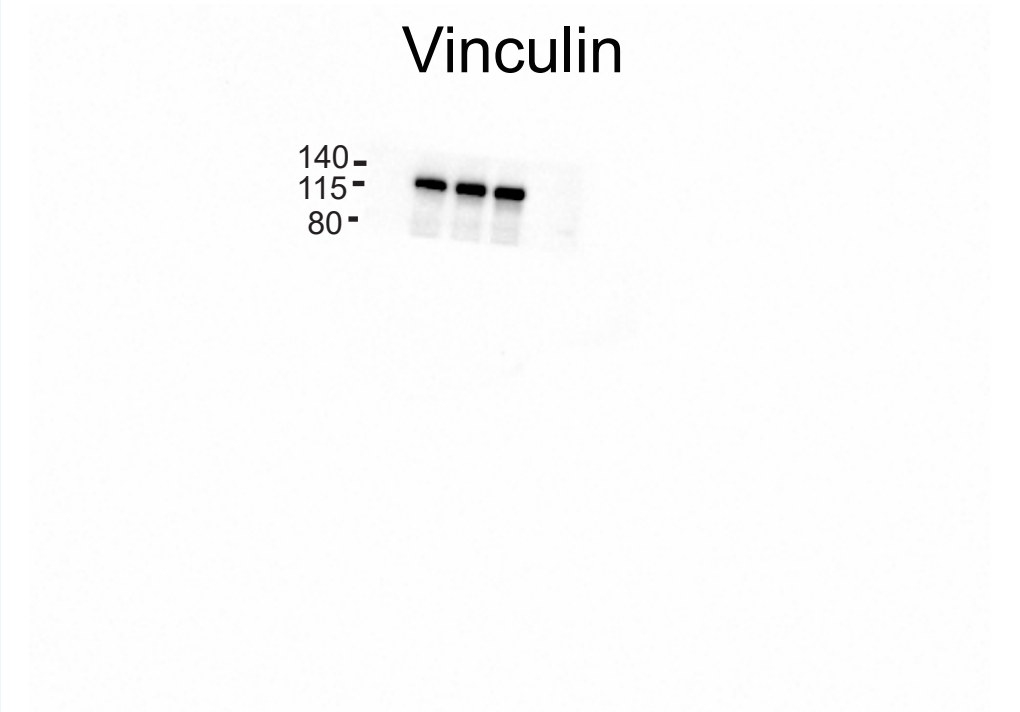

Vinculin WHITE

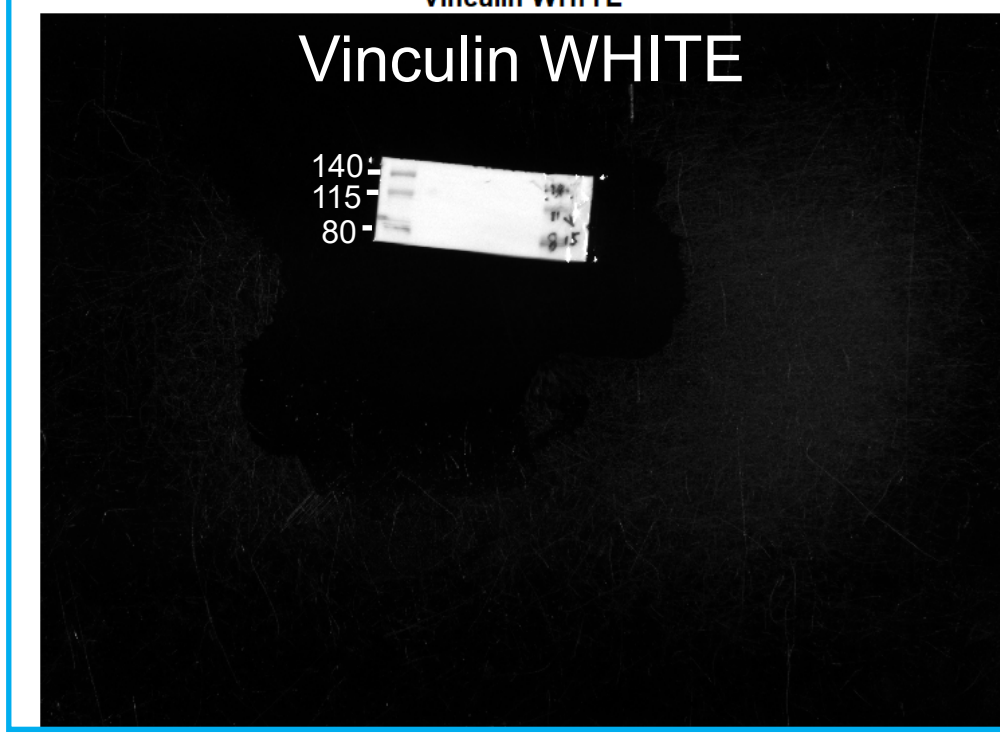

Total

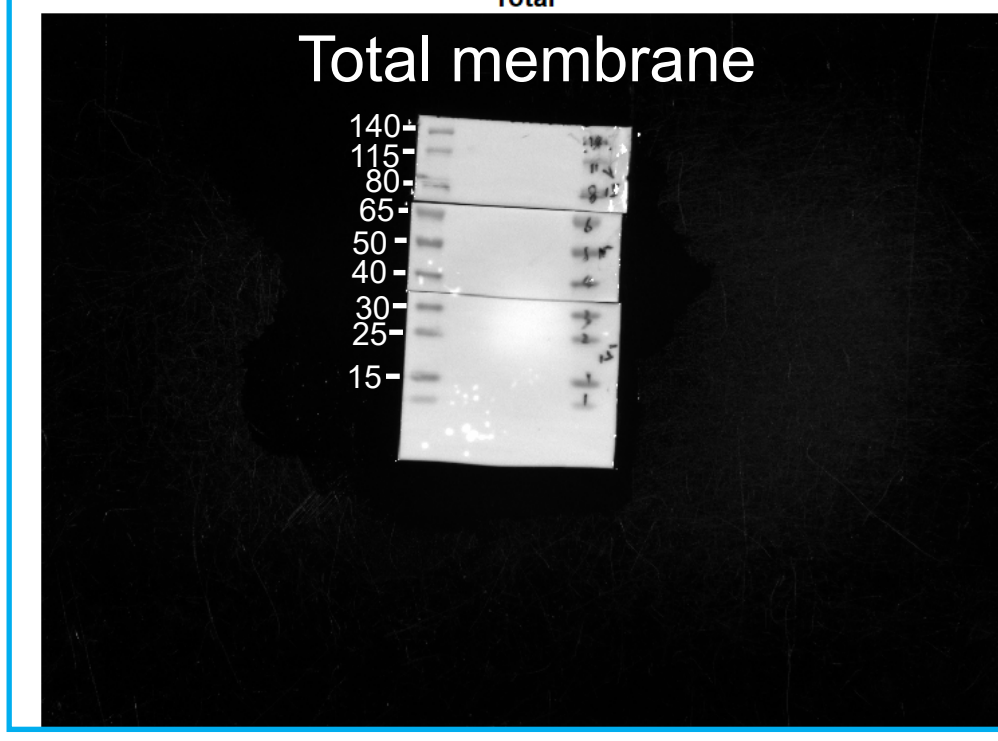

Supplement: Supplementary file 2 [file peerj-14-21763-s007.pdf]
