# Supplementary material for: Role of METTL6 in gastric cancer: diagnostic, prognostic, and functional implications
Source: PeerJ. 2026 Nov 2;14:e21763. doi: 10.7717/peerj.21763 (PMC13738193; doi:10.7717/peerj.21763)
Supplement: Supplementary file 4 [file peerj-14-21763-s003.docx]

| **Table S2.** The forest plot summarizing the predictive value on METTL6 expression in patients with GC according to different immune cell subsets | | | |
| --- | --- | --- | --- |
| Subgroup | Total(N) | HR (95% CI) | P value |
| Basophils |  |  |  |
| enriched | 66 | 3.09(0.72-13.27) | 0.11 |
| decreased | 303 | 1.67(1.1-2.52) | 0.015 |
| B-cells |  |  |  |
| enriched | 204 | 1.61(0.94-2.76 | 0.08 |
| decreased | 165 | 1.89(1.04-3.42) | 0.034 |
| CD4+ memory T-cells |  |  |  |
| enriched | 222 | 1.87(1.05-3.34) | 0.032 |
| decreased | 147 | 1.64(0.95-2.84) | 0.074 |
| CD8+ memory T-cells |  |  |  |
| enriched | 186 | 1.73(1.01-2.96) | 0.045 |
| decreased | 183 | 1.43(0.88-2.33) | 0.15 |
| Macrophages |  |  |  |
| enriched | 193 | 1.47(0.93-2.33) | 0.097 |
| decreased | 176 | 0.7(1.14-3.59 | 0.014 |
| Eosinophils |  |  |  |
| enriched | 277 | 1.8(1.15-2.83) | 0.009 |
| decreased | 92 | 1.43(0.72-2.83) | 0.3 |
| Mesenchymal stem cells | |  |  |
| enriched | 150 | 0.62(0.36-1.05) | 0.07 |
| decreased | 219 | 2.35(1.45-3.81) | 0.00037 |
| Natural killer T-cells |  |  |  |
| enriched | 237 | 1.91(1.13-3.25) | 0.015 |
| decreased | 132 | 0.56(0.29-1.09) | 0.082 |
| Regulatory T-cells |  |  |  |
| enriched | 272 | 1.71(1.15-2.54) | 0.0073 |
| decreased | 97 | 1.5(0.65-3.54) | 0.34 |
| Type 1 T-helper cells |  |  |  |
| enriched | 135 | 0.71(0.42-1.2) | 0.2 |
| decreased | 214 | 2.3(1.36-3.88) | 0.0013 |
| Type 2 T-helper cells |  |  |  |
| enriched | 344 | 1.8(1.18-2.73) | 0.0053 |
| decreased | 25 | 0.61(0.15-2.44) | 0.48 |
