# Supplementary material for: Role of METTL6 in gastric cancer: diagnostic, prognostic, and functional implications
Source: PeerJ. 2026 Nov 2;14:e21763. doi: 10.7717/peerj.21763 (PMC13738193; doi:10.7717/peerj.21763)
Supplement: Supplementary file 5 [file peerj-14-21763-s004.docx]

Table S3. Overview of statistical methods and R packages utilized for bioinformatic analyses.

| Figure Panel | Analysis Target | Statistical Test / Algorithm | R Package [Version] | Core Function / Execution |
| --- | --- | --- | --- | --- |
| Fig. 1A, 1B | Pan-cancer & Unpaired Tumor vs. Normal Expression | Wilcoxon rank-sum test | stats [4.2.1] | Comparison of continuous data distribution across two independent groups; log2(TPM + 1) transformed. |
|  |  |  | car [3.1-0] |  |
|  |  |  | ggplot2 [3.4.4] |  |
| Fig. 1C | Paired Tumor vs. Adjacent Normal Expression | Paired sample t-test | stats [4.2.1] | Evaluation of expression differences within matched sample pairs. |
|  |  |  | car [3.1-0] |  |
| Fig. 1D | Diagnostic Performance | ROC Curve & AUC Calculation | pROC [1.18.0] | Evaluation of sensitivity, specificity, and area under the curve (AUC). |
|  |  |  | ggplot2 [3.4.4] |  |
| Fig. 2A, 2B, 2I | Clinical Correlation (Gender, Age, OS Event) | Wilcoxon rank-sum test | stats [4.2.1] | Two-group comparison of METTL6 expression across clinical binary strata. |
|  |  |  | car [3.1-0] |  |
| Fig. 2C | Clinical Correlation (T stage) | One-way ANOVA | stats [4.2.1] | Multi-group parametric comparison of expression variance. |
|  |  |  | car [3.1-0] |  |
| Fig. 2D–2H | Clinical Correlation (N, M, Stage, Grade, Residual) | Kruskal-Wallis test | stats [4.2.1] | Non-parametric multi-group variance assessment for ordinal clinical stages. |
|  |  |  | car [3.1-0] |  |
| Fig. 3F–3H | Survival Analysis (OS, DSS, PFI) | Kaplan-Meier (Log-rank test) & Univariate Cox Regression | survival [3.3.1] | Time-to-event analysis stratified by median split (0–50% vs. 50–100%); assessment of proportional hazards assumption (cox.zph). |
|  |  |  | survminer [0.4.9] |  |
|  |  |  | ggplot2 [3.4.4] |  |
| Fig. 3I–3K | Prognostic Subgroup Analysis (N2, M1, CR primary therapy) | Kaplan-Meier (Log-rank test) & Univariate Cox Regression | survival [3.3.1] | Subgroup-specific survival modeling using stratified clinical filters. |
|  |  |  | survminer [0.4.9] |  |
| Fig. 6H | Immune Infiltration Correlation | ssGSEA Algorithm & Spearman Correlation | GSVA [1.46.0] | Calculation of 24 immune cell enrichment scores (ssGSEA) and correlation with METTL6 levels. |
|  |  |  | ggplot2 [3.4.4] |  |
| Fig. 6I | Immune Infiltration Group Comparison | ssGSEA Algorithm & Student's t-test | GSVA [1.46.0] | Identification of immune cell score shifts between high- and low-METTL6 expression cohorts. |
|  |  |  | stats [4.2.1] |  |
|  |  |  | car [3.1-0] |  |
