# Supplementary material for: Role of METTL6 in gastric cancer: diagnostic, prognostic, and functional implications
Source: PeerJ. 2026 Nov 2;14:e21763. doi: 10.7717/peerj.21763 (PMC13738193; doi:10.7717/peerj.21763)
Supplement: Supplementary file 6 [file peerj-14-21763-s008.docx]

**Clone formation**

| ShNC | ShMETTL6-1 | ShMETTL6-1 |
| --- | --- | --- |
| 129 | 56 | 54 |
| 166 | 51 | 49 |
| 137 | 38 | 56 |

Oneway

| **ANOVA** | | | | | |
| --- | --- | --- | --- | --- | --- |
| VAR00002 | | | | | |
|  | Sum of Squares | df | Mean Square | F | Sig. |
| Between Groups | 17454.889 | 2 | 8727.444 | 54.737 | .000 |
| Within Groups | 956.667 | 6 | 159.444 |  |  |
| Total | 18411.556 | 8 |  |  |  |

Post Hoc Tests

| **Multiple Comparisons** | | | | | | | | |
| --- | --- | --- | --- | --- | --- | --- | --- | --- |
| Dependent Variable: VAR00002 | | | | | | | | |
|  | (I) goup | (J) goup | Mean Difference (I-J) | Std. Error | Sig. | 95% Confidence Interval | |  |
|  |  |  |  |  |  | Lower Bound | Upper Bound |  |
| LSD | 1.00 | 2.00 | 95.66667^*^ | 10.31001 | .000 | 70.4390 | 120.8944 |  |
|  |  | 3.00 | 91.00000^*^ | 10.31001 | .000 | 65.7723 | 116.2277 |  |
|  | 2.00 | 1.00 | -95.66667^*^ | 10.31001 | .000 | -120.8944 | -70.4390 |  |
|  |  | 3.00 | -4.66667 | 10.31001 | .667 | -29.8944 | 20.5610 |  |
|  | 3.00 | 1.00 | -91.00000^*^ | 10.31001 | .000 | -116.2277 | -65.7723 |  |
|  |  | 2.00 | 4.66667 | 10.31001 | .667 | -20.5610 | 29.8944 |  |
| Tamhane | 1.00 | 2.00 | 95.66667^*^ | 12.45436 | .016 | 33.1228 | 158.2105 |  |
|  |  | 3.00 | 91.00000^*^ | 11.43095 | .038 | 11.8794 | 170.1206 |  |
|  | 2.00 | 1.00 | -95.66667^*^ | 12.45436 | .016 | -158.2105 | -33.1228 |  |
|  |  | 3.00 | -4.66667 | 5.75423 | .864 | -36.5232 | 27.1899 |  |
|  | 3.00 | 1.00 | -91.00000^*^ | 11.43095 | .038 | -170.1206 | -11.8794 |  |
|  |  | 2.00 | 4.66667 | 5.75423 | .864 | -27.1899 | 36.5232 |  |
| *. The mean difference is significant at the 0.05 level. | | | | | | | | |

1.00 means ShNC, 2.00 means shMETTL6-1, and 3 means shMETTL6-2.

**Wound-healing**

| Group | 0h distance | 48h distance | Migration rate(%) |
| --- | --- | --- | --- |
| ShNC-1 | 12.5676 | 5.7043 | 54.61106337 |
| ShNC-2 | 12.9561 | 6.5916 | 49.12357885 |
| ShNC-3 | 11.8829 | 4.9211 | 58.58670863 |
| ShMETTL6-1-1 | 12.2189 | 8.5472 | 10.61152502 |
| ShMETTL6-1-2 | 12.6438 | 11.3021 | 30.04934978 |
| ShMETTL6-1-3 | 12.2243 | 9.3916 | 23.17269701 |
| ShMETTL6-2-1 | 12.2179 | 8.6263 | 29.39621375 |
| ShMETTL6-2-2 | 12.3219 | 9.6961 | 21.31002524 |
| ShMETTL6-2-3 | 12.0486 | 9.9081 | 17.76554952 |

Oneway

| **ANOVA** | | | | | |
| --- | --- | --- | --- | --- | --- |
| VAR00002 | | | | | |
|  | Sum of Squares | df | Mean Square | F | Sig. |
| Between Groups | 2058.788 | 2 | 1029.394 | 19.890 | .002 |
| Within Groups | 310.531 | 6 | 51.755 |  |  |
| Total | 2369.319 | 8 |  |  |  |

Post Hoc Tests

| **ultiple Comparisons** | | | | | | | | | |
| --- | --- | --- | --- | --- | --- | --- | --- | --- | --- |
| Dependent Variable: VAR00002 | | | | | | | | |  |
|  | (I) group | (J) goup | Mean Difference (I-J) | Std. Error | Sig. | 95% Confidence Interval | |  |  |
|  |  |  |  |  |  | Lower Bound | Upper Bound |  |  |
| LSD | 1.00 | 2.00 | 32.82926^*^ | 5.87396 | .001 | 18.4562 | 47.2023 |  |  |
|  |  | 3.00 | 31.28319^*^ | 5.87396 | .002 | 16.9101 | 45.6562 |  |  |
|  | 2.00 | 1.00 | -32.82926^*^ | 5.87396 | .001 | -47.2023 | -18.4562 |  |  |
|  |  | 3.00 | -1.54607 | 5.87396 | .801 | -15.9191 | 12.8270 |  |  |
|  | 3.00 | 1.00 | -31.28319^*^ | 5.87396 | .002 | -45.6562 | -16.9101 |  |  |
|  |  | 2.00 | 1.54607 | 5.87396 | .801 | -12.8270 | 15.9191 |  |  |
| Tamhane | 1.00 | 2.00 | 32.82926^*^ | 6.31739 | .045 | 1.2593 | 64.3992 |  |  |
|  |  | 3.00 | 31.28319^*^ | 4.40133 | .007 | 13.4262 | 49.1402 |  |  |
|  | 2.00 | 1.00 | -32.82926^*^ | 6.31739 | .045 | -64.3992 | -1.2593 |  |  |
|  |  | 3.00 | -1.54607 | 6.65049 | .995 | -31.3690 | 28.2768 |  |  |
|  | 3.00 | 1.00 | -31.28319^*^ | 4.40133 | .007 | -49.1402 | -13.4262 |  |  |
|  |  | 2.00 | 1.54607 | 6.65049 | .995 | -28.2768 | 31.3690 |  |  |
| *. The mean difference is significant at the 0.05 level. | | | | | | | | | |

1.00 means ShNC, 2.00 means shMETTL6-1, and 3 means shMETTL6-2.

**Migration of Transwell**

| ShNC | ShMETTL6-1 | ShMETTL6-1 |
| --- | --- | --- |
| 179 | 48 | 44 |
| 254 | 89 | 94 |
| 186 | 84 | 66 |

Oneway

| **ANOVA** | | | | | |
| --- | --- | --- | --- | --- | --- |
|  | Sum of Squares | df | Mean Square | F | Sig. |
| Between Groups | 36768.667 | 2 | 18384.333 | 19.388 | .002 |
| Within Groups | 5689.333 | 6 | 948.222 |  |  |
| Total | 42458.000 | 8 |  |  |  |

Post Hoc Tests

| **ultiple Comparisons** | | | | | | | | |
| --- | --- | --- | --- | --- | --- | --- | --- | --- |
| Dependent Variable: VAR00002 | | | | | | | | |
|  | (I) Group | (J) group | Mean Difference (I-J) | Std. Error | Sig. | 95% Confidence Interval | |  |
|  |  |  |  |  |  | Lower Bound | Upper Bound |  |
| LSD | 1.00 | 2.00 | 132.66667^*^ | 25.14256 | .002 | 71.1450 | 194.1883 |  |
|  |  | 3.00 | 138.33333^*^ | 25.14256 | .002 | 76.8117 | 199.8550 |  |
|  | 2.00 | 1.00 | -132.66667^*^ | 25.14256 | .002 | -194.1883 | -71.1450 |  |
|  |  | 3.00 | 5.66667 | 25.14256 | .829 | -55.8550 | 67.1883 |  |
|  | 3.00 | 1.00 | -138.33333^*^ | 25.14256 | .002 | -199.8550 | -76.8117 |  |
|  |  | 2.00 | -5.66667 | 25.14256 | .829 | -67.1883 | 55.8550 |  |
| Tamhane | 1.00 | 2.00 | 132.66667^*^ | 27.18251 | .046 | 4.1260 | 261.2073 |  |
|  |  | 3.00 | 138.33333^*^ | 27.95433 | .038 | 12.9852 | 263.6815 |  |
|  | 2.00 | 1.00 | -132.66667^*^ | 27.18251 | .046 | -261.2073 | -4.1260 |  |
|  |  | 3.00 | 5.66667 | 19.39358 | .990 | -71.3274 | 82.6608 |  |
|  | 3.00 | 1.00 | -138.33333^*^ | 27.95433 | .038 | -263.6815 | -12.9852 |  |
|  |  | 2.00 | -5.66667 | 19.39358 | .990 | -82.6608 | 71.3274 |  |
| *. The mean difference is significant at the 0.05 level. | | | | | | | | |

1.00 means ShNC, 2.00 means shMETTL6-1, and 3 means shMETTL6-2.

**Invasion of Transwell**

| ShNC | ShMETTL6-1 | ShMETTL6-1 |
| --- | --- | --- |
| 111 | 29 | 29 |
| 149 | 37 | 37 |
| 114 | 39 | 36 |

Oneway

| **ANOVA** | | | | | |
| --- | --- | --- | --- | --- | --- |
| VAR00002 | | | | | |
|  | Sum of Squares | df | Mean Square | F | Sig. |
| Between Groups | 16261.556 | 2 | 8130.778 | 49.444 | .000 |
| Within Groups | 986.667 | 6 | 164.444 |  |  |
| Total | 17248.222 | 8 |  |  |  |

Post Hoc Tests

| **Multiple Comparisons** | | | | | | | | |
| --- | --- | --- | --- | --- | --- | --- | --- | --- |
| Dependent Variable: VAR00002 | | | | | | | | |
|  | (I) group | (J) group | Mean Difference (I-J) | Std. Error | Sig. | 95% Confidence Interval | |  |
|  |  |  |  |  |  | Lower Bound | Upper Bound |  |
| LSD | 1.00 | 2.00 | 89.66667^*^ | 10.47042 | .000 | 64.0465 | 115.2869 |  |
|  |  | 3.00 | 90.66667^*^ | 10.47042 | .000 | 65.0465 | 116.2869 |  |
|  | 2.00 | 1.00 | -89.66667^*^ | 10.47042 | .000 | -115.2869 | -64.0465 |  |
|  |  | 3.00 | 1.00000 | 10.47042 | .927 | -24.6202 | 26.6202 |  |
|  | 3.00 | 1.00 | -90.66667^*^ | 10.47042 | .000 | -116.2869 | -65.0465 |  |
|  |  | 2.00 | -1.00000 | 10.47042 | .927 | -26.6202 | 24.6202 |  |
| Tamhane | 1.00 | 2.00 | 89.66667^*^ | 12.57422 | .041 | 8.1560 | 171.1773 |  |
|  |  | 3.00 | 90.66667^*^ | 12.45436 | .043 | 6.1639 | 175.1694 |  |
|  | 2.00 | 1.00 | -89.66667^*^ | 12.57422 | .041 | -171.1773 | -8.1560 |  |
|  |  | 3.00 | 1.00000 | 3.95811 | .994 | -14.9356 | 16.9356 |  |
|  | 3.00 | 1.00 | -90.66667^*^ | 12.45436 | .043 | -175.1694 | -6.1639 |  |
|  |  | 2.00 | -1.00000 | 3.95811 | .994 | -16.9356 | 14.9356 |  |
| *. The mean difference is significant at the 0.05 level. | | | | | | | | |

1.00 means ShNC, 2.00 means shMETTL6-1, and 3 means shMETTL6-2.

**Tumor Volume**

|  | Day 0 | | | Day 7 | | | Day 14 | | | Day 21 | | | Day 28 | | |
| --- | --- | --- | --- | --- | --- | --- | --- | --- | --- | --- | --- | --- | --- | --- | --- |
| Group | LD | SD | V | LD | SD | V | LD | SD | V | LD | SD | V | LD | SD | V |
| Control | 6.11 | 6.01 | 110.3469 | 8.18 | 8.08 | 267.0214 | 9.38 | 9.28 | 403.8953 | 11.74 | 10.17 | 607.1276 | 14.02 | 11.29 | 893.5233 |
| Control | 8.6 | 4.83 | 100.3143 | 9.08 | 8.28 | 311.2551 | 11.39 | 9.03 | 464.3754 | 12.73 | 8.88 | 501.9083 | 14.63 | 10.52 | 809.554 |
| Control | 7.18 | 5.45 | 106.632 | 8.78 | 7.3 | 233.9431 | 9.14 | 8.5 | 330.1825 | 12.82 | 9.73 | 606.8533 | 14.75 | 10.24 | 773.3248 |
| Control | 7.57 | 5.46 | 112.8369 | 7.79 | 7.69 | 230.3351 | 10.08 | 9.65 | 469.3374 | 10.47 | 9.02 | 425.9217 | 12.33 | 10.83 | 723.0861 |
| Control | 6.97 | 4.68 | 76.32986 | 7.12 | 6.71 | 160.2858 | 10.07 | 9.39 | 443.9465 | 10.11 | 8.01 | 324.3293 | 11.1 | 10.07 | 562.7972 |
| Control | 6.78 | 6.21 | 130.7323 | 7.63 | 7.13 | 193.9428 | 8.56 | 8.12 | 282.1992 | 10.87 | 9.88 | 530.5343 | 12.05 | 11.32 | 772.058 |
| sh1 | 6.24 | 5.55 | 96.1038 | 7.55 | 6.45 | 157.0494 | 8.87 | 7.77 | 267.7538 | 9.86 | 8.76 | 378.3164 | 10.32 | 9.22 | 438.6433 |
| sh1 | 8.05 | 4.39 | 77.5702 | 9.75 | 6.11 | 181.994 | 8.98 | 7.31 | 239.9281 | 8.58 | 8.08 | 280.0787 | 9.51 | 9.01 | 386.0114 |
| sh1 | 6.22 | 5.63 | 98.57736 | 7.42 | 6.32 | 148.1863 | 8.18 | 7.08 | 205.017 | 9.76 | 8.35 | 340.2458 | 11.35 | 9.29 | 489.7758 |
| sh1 | 6.63 | 5.28 | 92.4169 | 6.78 | 6.68 | 151.2699 | 7.03 | 6.93 | 168.8075 | 8.42 | 8.32 | 291.4263 | 10.61 | 10.51 | 585.9908 |
| sh1 | 6.21 | 5.92 | 108.8191 | 6.93 | 6.83 | 161.6384 | 7.83 | 7.73 | 233.9326 | 8.8 | 8.15 | 292.259 | 10.39 | 8.07 | 338.3239 |
| sh1 | 7.69 | 6.58 | 166.4747 | 7.32 | 7.13 | 186.0631 | 8.55 | 7.93 | 268.8329 | 9.62 | 8.31 | 332.1598 | 11.53 | 8.12 | 380.1118 |
| sh2 | 7.63 | 5.29 | 106.7593 | 9.79 | 6.3 | 194.2826 | 9.25 | 7.36 | 250.5344 | 10.84 | 9.87 | 527.9996 | 11.9 | 10.1 | 606.9595 |
| sh2 | 7.99 | 4.6 | 84.5342 | 8.32 | 6.22 | 160.9437 | 8.89 | 8.86 | 348.9307 | 9.78 | 9.34 | 426.5821 | 10.75 | 9.43 | 477.9713 |
| sh2 | 8.09 | 5.42 | 118.8275 | 9.82 | 7.14 | 250.3098 | 9.96 | 7.93 | 313.1668 | 9.81 | 8.07 | 319.4376 | 10.36 | 8.26 | 353.419 |
| sh2 | 8.47 | 4.61 | 90.00264 | 8.64 | 6.54 | 184.7733 | 9.28 | 7.09 | 233.244 | 10.87 | 7.35 | 293.6123 | 9.47 | 8.83 | 369.1827 |
| sh2 | 9.99 | 4.4 | 96.7032 | 10.45 | 6.45 | 217.3731 | 10.79 | 7.68 | 318.21 | 9.74 | 7.94 | 307.0223 | 10.1 | 9.01 | 409.9595 |
| sh2 | 7.36 | 6.44 | 152.6228 | 8.25 | 7.12 | 209.1144 | 7.69 | 7.13 | 195.4679 | 8.25 | 7.64 | 240.7746 | 8.67 | 8.13 | 286.5301 |

Sh1 means shMETTL6-1, sh2 means shMETTL6-2, LD means LD (mm), SD means SD (mm), V means volume (mm3)

Oneway

| **ANOVA** | | | | | |
| --- | --- | --- | --- | --- | --- |
| VAR00002 | | | | | |
|  | Sum of Squares | df | Mean Square | F | Sig. |
| Between Groups | 433582.183 | 2 | 216791.092 | 19.767 | .000 |
| Within Groups | 164509.033 | 15 | 10967.269 |  |  |
| Total | 598091.216 | 17 |  |  |  |

Post Hoc Tests

| **Multiple Comparisons** | | | | | | | | |
| --- | --- | --- | --- | --- | --- | --- | --- | --- |
| Dependent Variable: VAR00002 | | | | | | | | |
|  | (I) group | (J) group | Mean Difference (I-J) | Std. Error | Sig. | 95% Confidence Interval | |  |
|  |  |  |  |  |  | Lower Bound | Upper Bound |  |
| LSD | 1.00 | 2.00 | 319.24773^*^ | 60.46285 | .000 | 190.3742 | 448.1212 |  |
|  |  | 3.00 | 338.38687^*^ | 60.46285 | .000 | 209.5134 | 467.2604 |  |
|  | 2.00 | 1.00 | -319.24773^*^ | 60.46285 | .000 | -448.1212 | -190.3742 |  |
|  |  | 3.00 | 19.13915 | 60.46285 | .756 | -109.7344 | 148.0127 |  |
|  | 3.00 | 1.00 | -338.38687^*^ | 60.46285 | .000 | -467.2604 | -209.5134 |  |
|  |  | 2.00 | -19.13915 | 60.46285 | .756 | -148.0127 | 109.7344 |  |
| Tamhane | 1.00 | 2.00 | 319.24773^*^ | 58.11098 | .001 | 151.7538 | 486.7417 |  |
|  |  | 3.00 | 338.38687^*^ | 64.27839 | .001 | 154.5268 | 522.2469 |  |
|  | 2.00 | 1.00 | -319.24773^*^ | 58.11098 | .001 | -486.7417 | -151.7538 |  |
|  |  | 3.00 | 19.13915 | 58.81047 | .985 | -150.6323 | 188.9106 |  |
|  | 3.00 | 1.00 | -338.38687^*^ | 64.27839 | .001 | -522.2469 | -154.5268 |  |
|  |  | 2.00 | -19.13915 | 58.81047 | .985 | -188.9106 | 150.6323 |  |
| *. The mean difference is significant at the 0.05 level. | | | | | | | | |

1.00 means ShNC, 2.00 means shMETTL6-1, and 3 means shMETTL6-2.

**Tumor weight**

| ShNC | ShMETTL6-1 | ShMETTL6-1 |
| --- | --- | --- |
| 0.979 | 0.551 | 0.544 |
| 0.886 | 0.485 | 0.559 |
| 0.798 | 0.665 | 0.488 |
| 0.704 | 0.656 | 0.442 |
| 0.787 | 0.485 | 0.391 |
| 0.807 | 0.408 | 0.259 |

Oneway

| ANOVA | | | | | |
| --- | --- | --- | --- | --- | --- |
| VAR00002 | | | | | |
|  | Sum of Squares | df | Mean Square | F | Sig. |
| Between Groups | .469 | 2 | .234 | 22.064 | .000 |
| Within Groups | .159 | 15 | .011 |  |  |
| Total | .628 | 17 |  |  |  |

Post Hoc Tests

| Multiple Comparisons | | | | | | | |
| --- | --- | --- | --- | --- | --- | --- | --- |
| Dependent Variable: VAR00002 | | | | | | | |
|  | (I) group | (J) group | Mean Difference (I-J) | Std. Error | Sig. | 95% Confidence Interval | |
|  |  |  |  |  |  | Lower Bound | Upper Bound |
| LSD | 1.00 | 2.00 | .28517^*^ | .05951 | .000 | .1583 | .4120 |
|  |  | 3.00 | .37967^*^ | .05951 | .000 | .2528 | .5065 |
|  | 2.00 | 1.00 | -.28517^*^ | .05951 | .000 | -.4120 | -.1583 |
|  |  | 3.00 | .09450 | .05951 | .133 | -.0323 | .2213 |
|  | 3.00 | 1.00 | -.37967^*^ | .05951 | .000 | -.5065 | -.2528 |
|  |  | 2.00 | -.09450 | .05951 | .133 | -.2213 | .0323 |
| Tamhane | 1.00 | 2.00 | .28517^*^ | .05693 | .002 | .1221 | .4482 |
|  |  | 3.00 | .37967^*^ | .05964 | .000 | .2082 | .5511 |
|  | 2.00 | 1.00 | -.28517^*^ | .05693 | .002 | -.4482 | -.1221 |
|  |  | 3.00 | .09450 | .06186 | .403 | -.0827 | .2717 |
|  | 3.00 | 1.00 | -.37967^*^ | .05964 | .000 | -.5511 | -.2082 |
|  |  | 2.00 | -.09450 | .06186 | .403 | -.2717 | .0827 |
| *. The mean difference is significant at the 0.05 level. | | | | | | | |

1.00 means ShNC, 2.00 means shMETTL6-1, and 3 means shMETTL6-2.
