# Supplementary material for: Role of METTL6 in gastric cancer: diagnostic, prognostic, and functional implications
Source: PeerJ. 2026 Nov 2;14:e21763. doi: 10.7717/peerj.21763 (PMC13738193; doi:10.7717/peerj.21763)
Supplement: Supplementary file 7 [file peerj-14-21763-s002.doc]

**Table S1**. Tumor volumes in the subcutaneous xenograft model.

| Group | day0 | | | day7 | | | day14 | | | day21 | | | day28 | | |
| --- | --- | --- | --- | --- | --- | --- | --- | --- | --- | --- | --- | --- | --- | --- | --- |
|  | Long | Short | Volume | Long | Short | Volume | Long | Short | Volume | Long | Short | Volume | Long | Short | Volume |
| Control | 6.15 | 6.09 | 114.0459 | 7.51 | 6.16 | 142.4857 | 9.37 | 8.02 | 301.3411 | 12.5 | 10.06 | 632.5225 | 15.11 | 11.72 | 1037.743 |
|  | 7 | 5.76 | 116.1216 | 9.26 | 6.69 | 207.2207 | 10.97 | 8.4 | 387.0216 | 13.93 | 9.94 | 688.1671 | 15.61 | 11.98 | 1120.177 |
|  | 8.06 | 4.88 | 95.97203 | 8 | 7.78 | 242.1136 | 9.21 | 8.99 | 372.1766 | 11.52 | 10.3 | 611.0784 | 14.86 | 11.61 | 1001.505 |
|  | 7.69 | 6.17 | 146.3749 | 9.93 | 6.73 | 224.8792 | 11.12 | 7.92 | 348.7588 | 12.63 | 9.83 | 610.2115 | 13.78 | 11.25 | 872.0156 |
|  | 8.38 | 4.57 | 87.50773 | 9.11 | 5.79 | 152.7023 | 10.56 | 8.24 | 358.4993 | 13.22 | 8.99 | 534.2209 | 13.15 | 10.88 | 778.3117 |
|  | 7.87 | 4.51 | 80.03829 | 10.65 | 5.99 | 191.0615 | 13.38 | 7.72 | 398.7133 | 15.07 | 9.11 | 625.3455 | 16.03 | 10.12 | 820.8514 |
| Sh-1 | 7.71 | 5.19 | 103.8387 | 9.51 | 6.35 | 191.7335 | 10.78 | 7.62 | 312.9671 | 11.26 | 8.8 | 435.9872 | 12.79 | 9.98 | 636.9446 |
|  | 6.13 | 4.91 | 73.89133 | 7.28 | 6.44 | 150.9639 | 8.34 | 7.5 | 234.5625 | 10.31 | 9.47 | 462.305 | 12.94 | 9.49 | 582.6888 |
|  | 7.05 | 4.87 | 83.60207 | 6.67 | 6.05 | 122.0693 | 7.93 | 7.31 | 211.8741 | 9.4 | 8.78 | 362.3155 | 12.53 | 8.99 | 506.3379 |
|  | 7.28 | 6.12 | 136.334 | 7.54 | 5.47 | 112.8018 | 8.09 | 6.52 | 171.9546 | 9.76 | 8.19 | 327.3314 | 11.89 | 8.98 | 479.4072 |
|  | 8.24 | 5.13 | 108.4256 | 7.42 | 6.17 | 141.2356 | 8.3 | 7.05 | 206.2654 | 9.71 | 8.46 | 347.4801 | 11.54 | 9.69 | 541.7805 |
|  | 7.05 | 6.3 | 139.9073 | 8.06 | 6.78 | 185.2527 | 8.7 | 7.42 | 239.4953 | 10.47 | 9.19 | 442.1277 | 11.2 | 10.67 | 637.5538 |
| Sh-2 | 7.34 | 5.94 | 129.4908 | 9.47 | 6.54 | 202.5235 | 9.99 | 6.87 | 235.7485 | 12.9 | 8.28 | 442.2017 | 14.16 | 9.25 | 605.7825 |
|  | 8.19 | 5.13 | 107.7677 | 8.38 | 6.33 | 167.8887 | 8.91 | 7.54 | 253.2739 | 11.72 | 8.66 | 439.4742 | 14.09 | 9.26 | 604.0918 |
|  | 7.45 | 6.3 | 147.8453 | 7.89 | 6.93 | 189.4582 | 8.58 | 7.05 | 213.2237 | 10.67 | 8.36 | 372.861 | 13.3 | 9.02 | 541.0467 |
|  | 7.42 | 5.38 | 107.3837 | 7.97 | 5.89 | 138.248 | 8.65 | 6.93 | 207.7077 | 10.46 | 6.87 | 246.8398 | 12.67 | 8.46 | 453.4061 |
|  | 6.94 | 4.89 | 82.97499 | 7.53 | 5.08 | 97.1611 | 7.93 | 6.01 | 143.2162 | 9.002 | 7.16 | 230.7465 | 10.272 | 9.82 | 495.2768 |
|  | 6.91 | 4.67 | 75.34975 | 8.06 | 5.45 | 119.7011 | 8.87 | 5.94 | 156.4828 | 10.62 | 7.59 | 305.899 | 12.23 | 8.18 | 409.1693 |

Long = longest diameter (mm), Short = shortest diameter (mm), Volume (mm³).
